# Co-targeting CDK2 and CDK4/6 overcomes resistance to aromatase and CDK4/6 inhibitors in ER+ breast cancer

**DOI:** 10.1038/s41698-022-00311-6

**Published:** 2022-09-24

**Authors:** Abeer J. Al-Qasem, Carla L. Alves, Sidse Ehmsen, Martina Tuttolomondo, Mikkel G. Terp, Lene E. Johansen, Henriette Vever, Luna V. A. Hoeg, Daniel Elias, Martin Bak, Henrik J. Ditzel

**Affiliations:** 1 Department of Cancer and Inflammation Research, Institute of Molecular Medicine, University of Southern Denmark, Odense, Denmark; 2 Department of Oncology, Odense University Hospital, Institute of Clinical Research, University of Southern Denmark, Odense, Denmark; 3 Department of Pathology, Sydvestjysk Sygehus, Esbjerg, Denmark; 4 Academy of Geriatric Cancer Research (AgeCare), Odense University Hospital, Odense, Denmark

**Keywords:** Targeted therapies, Predictive markers

## Abstract

Resistance to aromatase inhibitor (AI) treatment and combined CDK4/6 inhibitor (CDK4/6i) and endocrine therapy (ET) are crucial clinical challenges in treating estrogen receptor-positive (ER+) breast cancer. Understanding the resistance mechanisms and identifying reliable predictive biomarkers and novel treatment combinations to overcome resistance are urgently needed. Herein, we show that upregulation of CDK6, p-CDK2, and/or cyclin E1 is associated with adaptation and resistance to AI-monotherapy and combined CDK4/6i and ET in ER+ advanced breast cancer. Importantly, co-targeting CDK2 and CDK4/6 with ET synergistically impairs cellular growth, induces cell cycle arrest and apoptosis, and delays progression in AI-resistant and combined CDK4/6i and fulvestrant-resistant cell models and in an AI-resistant autocrine breast tumor in a postmenopausal xenograft model. Analysis of CDK6, p-CDK2, and/or cyclin E1 expression as a combined biomarker in metastatic lesions of ER+ advanced breast cancer patients treated with AI-monotherapy or combined CDK4/6i and ET revealed a correlation between high biomarker expression and shorter progression-free survival (PFS), and the biomarker combination was an independent prognostic factor in both patients cohorts. Our study supports the clinical development of therapeutic strategies co-targeting ER, CDK4/6 and CDK2 following progression on AI-monotherapy or combined CDK4/6i and ET to improve survival of patients exhibiting high tumor levels of CDK6, p-CDK2, and/or cyclin E1.

## Introduction

Aromatase inhibitors (AI) have proven clinical efficacy both in the adjuvant and advanced settings in estrogen receptor-positive (ER+) breast cancer (BC) patients^1–6^. This type of endocrine therapy (ET) blocks the enzymatic conversion of intra-tumoral and non-ovarian circulating androgens to estrogen^1–3^. Despite their proven activity, the development of endocrine resistance remains a major clinical challenge^7–9^. Importantly, the key role of the cyclin D-CDK4/6-Rb cascade, which regulates the early G1/S cell cycle transition, in ER+ BC tumorigenesis and endocrine resistance is well-known^10–14^. Furthermore, cyclin D-CDK4/6-Rb pathway is downstream of multiple oncogenic pathways, including ER and PI3K/AKT/mTOR signaling^10^. Targeting this cell cycle cascade was found to be critical to overcome endocrine resistance and supported the clinical investigation of CDK4/6 inhibitors (CDK4/6i; palbociclib, ribociclib, and abemaciclib) in combination with ET in ER+ BC. The findings of these clinical trials led to the approval of CDK4/6i in combination with ET AI or fulvestrant (selective estrogen receptor down-regulator), which is the current first-line treatment in advanced BC (ABC)^15–21^. However, it remains uncertain whether AI or fulvestrant should be the preferred ET backbone in combination with CDK4/6i, as the two combinations have not been directly compared. Despite the substantial improvement of progression-free survival (PFS) and overall survival (OS) with combined CDK4/6i and ET, resistance remains a major clinical challenge for this treatment option. Approximately 20% of patients exhibit primary resistance, while acquired resistance eventually develops for all patients. Accordingly, there is an urgent need to identify targetable predictive biomarkers to improve patient selection and develop novel biomarker-driven treatment strategies to benefit these patients.

Clinically, ER remains the only currently approved biomarker of response to combined CDK4/6i and ET treatment, and biomarkers predicting resistance are not available^22^. Several resistance mechanisms to combined CDK4/6i and ET have been suggested, some of which are shared with those following progression on ET-monotherapy, such as *PIK3CA* and *ESR1* mutations and upregulation of CDK6, as shown by retrospective analysis of tumors in the PALOMA-3 study^17,23–25^. This association implicates ET as a major driver of resistance to the sequential combined treatment incorporating CDK4/6i. Although this subject has been extensively investigated, underlying mechanisms mediating endocrine resistance are still poorly understood.

Recent studies have demonstrated a role of the cyclin E-CDK2-Rb cascade, which regulates the late G1/S cell cycle transition, in BC tumorigenesis and endocrine resistance^26–28^. Several preclinical investigations have reported that an oncogenic, low molecular weight, isoform of cyclin E1 (LMW-E; with cytoplasmic localization) mediates AI resistance in genetically engineered cell models (LMW-E/aromatase-induced cells)^26–28^. The importance of the overexpression/amplification of *CCNE1* (encodes cyclin E1) and non-canonical activation of cyclin D-CDK2 has also been suggested in preclinical studies using long-term CDK4/6i-resistant cell models, including in BC models^29–31^. Furthermore, loss of *RB1* has also been reported in CDK4/6i-resistant preclinical studies^30,32,33^. However, analysis of AI-pretreated tumors in the PALOMA-3 trial demonstrated no significant association between these biomarkers and resistance to combined CDK4/6i and fulvestrant^34^.

In this study, we aimed to identify key molecules of early resistance to AI and combined CDK4/6i and ET focusing on alterations in the G1/S cell cycle transition cascades. This was done by investigating different cell models recapitulating the heterogeneity of developed resistance to AI and supported by cell models resistant to the combined CDK4/6i and fulvestrant treatment. The clinical relevance of these findings was further evaluated in animal models and clinical ABC samples.

Herein, we show that interrogating the dysregulation of both cyclin D-CDK4/6-Rb and cyclin E-CDK2-Rb G1/S transition cooperative cascades will provide insight into the efficacy of the addition of CDK4/6i in the treatment strategy upon AI progression.

## Results

### Gene expression analysis identifies upregulation of G1/S transition modulators in AI-resistant and combined CDK4/6i and fulvestrant-resistant ER+ BC cells

**Fig. 1:**
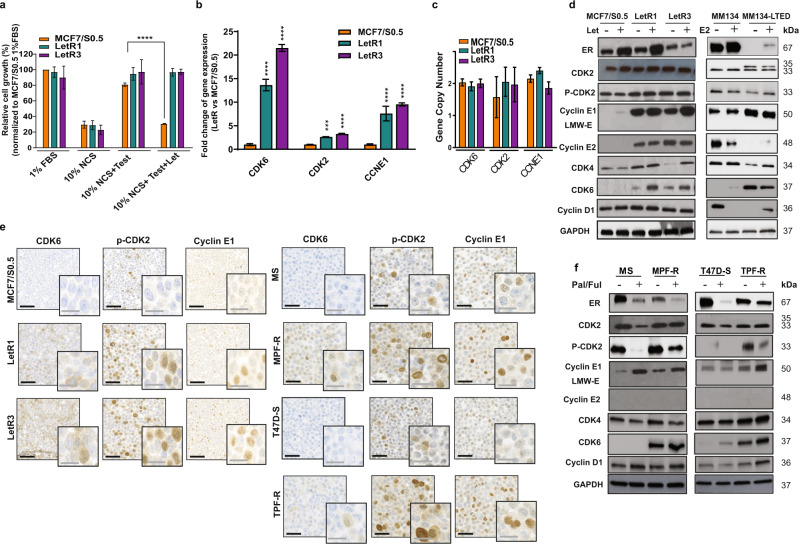
Altered expression of G1/S transition cooperative cascades cyclin D-CDK4/6-Rb and cyclin E-CDK2-Rb in AI-resistant and combined palbociclib and fulvestrant-resistant ER+ BC cells. **a** Evaluation of cell growth of letrozole-resistant (LetR) cells and the sensitive MCF7/S0.5 cell line by crystal violet colorimetric assay. Relative cell growth (%) of independent experiments in triplicates ± SD is shown. Statistically significant differences by two-way ANOVA are shown as ****p ≤ 0.0001. **b** Quantitative RT-PCR verifying the gene expression alteration of *CDK6*, *CDK2*, and *CCNE1* (encodes cyclin E1). The expression was normalized using *PUM1* gene and shown as relative expression in LetR vs. MCF7/S0.5 cells. **c** Quantitative RT-PCR copy-number assay using Taqman copy-number variants (CNV) primers. Average calculated copy-number values are plotted with bars representing values from replicate measurements (*n* = 4) ± SD. **d** Western blotting analysis of lysates from LetR and MCF7/S0.5 cells cultured at aromatase-dependent growth condition (10% NCS + 100 nM testosterone) in the presence or absence of letrozole (Let, 1 μM). Lysates from AI-resistant MM134-LTED cells and the sensitive MM134 cells treated with or without estradiol (E2, 1 μg/ml) for 4 days. GAPDH was used as loading control. A representative of two biological replicates is shown. **e** Micrographs of immunocytochemistry analysis of formalin-fixed paraffin-embedded (FFPE) AI-resistant or combined CDK4/6i and fulvestrant-resistant cells stained for CDK6, p-CDK2, and cyclin E1 (black scale bar: 100 µm, gray scale bar: 50 µm). **f** Western blotting analysis of lysates from the combined palbociclib and fulvetsrant-resistant cells (MPF-R and TPF-R) and the sensitive cell lines (MS and T47D-S, respectively) treated with or without CDK4/6i palbociclib (Pal, 150 nM) and fulvestrant (Ful, 100 nM) for 4 days. β-actin was used as loading control. A representative of two biological replicates is shown. Statistically significant differences calculated by one-way ANOVA are shown as ns *p* > 0.05, *p ≤ 0.05, ***p ≤ 0.001, and ****p ≤ 0.0001.

Initially, we evaluated the growth of the unique genetically unmodified ER+ BC cell models (LetR1 and LetR3), which mimic resistance to the AI letrozole due to local estrogen synthesis through aromatization of testosterone (aromatase-mediated growth condition)^35^. In both LetR cells and the parental-sensitive MCF7/S0 cell line, we observed comparable growth stimulation between aromatase-mediated growth conditions (10% newborn calf serum (NCS) and 100 nM testosterone) and exogenous supplied estrogen (1% FBS) (Fig. 1a). The withdrawal of testosterone from the aromatase-mediated growth condition (10% NCS) significantly reduced the growth of all cell lines (Fig. 1a). Thus, the growth of LetR cells is estrogen-dependent and these cells are able to aromatize testosterone for local estrogen synthesis, comparable to the parental MCF7/S0.5 cells. However, upon addition of letrozole, no reduction of cell growth was observed in LetR cells, while the parental MCF7/S0.5 cell growth was significantly inhibited, demonstrating the letrozole-resistance phenotype of LetR cells (Fig. 1a). Resistance to letrozole was not associated with androgen dependency, as sensitive and resistant cells showed comparable response to the androgen receptor inhibitor enzalutamide in either 1% FBS or 10% NCS with testosterone (Supplementary Fig. 1). Next, we performed gene array analysis to identify gene expression alterations associated with acquired resistance to letrozole and found a total of 1364 genes (690 upregulated and 674 downregulated) that exhibited significantly altered expression (fold-change ≥2, FDR < 0.01, one-way ANOVA *p* < 0.01) in LetR vs. MCF7/S0.5 cells. Importantly, alterations in the regulators of cell cycle phase transitions were identified as the most significantly enriched gene-datasets by Gene Set Enrichment Analyses (GSEA, Supplementary Fig. 2 and Supplementary Table 1). In particular, three G1/S transition modulators, CDK6, CDK2 and cyclin E1, exhibited significantly altered expression in LetR vs. MCF7/S0.5 cells. These genes are also consistently reported in the most significantly altered canonical pathways, including cell cycle control of chromosomal replication and estrogen-mediated S-phase entry, as determined by IPA (Supplementary Table 2). Moreover, these genes were also found to be implicated in the gene networks associated with cell cycle regulation and cancer-related mechanisms (Supplementary Table 2). Importantly, significant upregulation of these G1/S transition modulators was observed in the gene expression analysis of the individual LetR cells (LetR1 and LetR3) compared with the parental cell line, MCF7/S0.5 (Supplementary Table 3), and confirmed using quantitative RT-PCR (Fig. 1b). Notably, TaqMan-based copy-number analysis revealed no gene amplification for either *CDK6*, *CDK2* or *CCNE1* in LetR cells compared to MCF7/S0.5 cell line (Fig. 1c). Furthermore, we evaluated alteration of the cyclin D-CDK4/6-Rb and cyclin E-CDK2-Rb G1/S transition cooperative cascades at the protein level in both LetR and MCF7/S0.5 cell model and in another AI-resistant model including long-term estrogen-deprived (LTED) MM134-LTED cells and their sensitive MM134 cell line (Fig. 1d and Supplementary Fig. 3a). Upregulation of CDK6 and cyclin E1 was observed in both AI-resistant cells (LetR and MM134-LTED) compared with their parental cells (MCF7/S0.5 and MM134, respectively), in the presence of letrozole (LetR cells) or absence of estrogen (MM134-LTED cells), while upregulation of phospho-CDK2 (p-CDK2) was found only in LetR cells vs. MCF7/S0.5 cells. In contrast to recent studies, the low molecular weight form of cyclin E1 (LMW-E, cytoplasmic localization) was not observed to be associated with AI resistance (Fig. 1d)^26,27^. Instead, increased expression of the full-length form of cyclin E1 (nucleus localized) was detected by western blotting using the monoclonal anti-cyclin E antibody (clone HE12), which was previously reported to differentiate between the nuclear vs. cytoplasmic forms of cyclin E1^27^ (Fig. 1d). The altered protein expression was confirmed by immunocytochemical (ICC) staining in the LetR cell model (Fig. 1e).

We next examined whether the alteration of the G1/S transition cooperative cascades is also observed in the combined CDK4/6i (palbociclib) and fulvestrant-resistant ER+ BC cell model, which is the sequential treatment upon progression on AI-monotherapy. Interestingly, upregulation of CDK6, p-CDK2 and cyclin E1 was observed in T47D-derived-resistant cells (TPF-R) compared with the sensitive cell lines (T47D-S), and the MCF7-derived-resistant cells (MPF-R) showed also upregulation of CDK6 and p-CDK2 compared to the parental cell line (MS), as shown by ICC and western blotting (Fig. 1e, f and Supplementary Fig. 3b). These data suggest a common alteration of the cyclin D-CDK6-Rb and cyclin E-CDK2-Rb G1/S transition modulators in the resistance to AI-monotherapy and the sequential combination treatment of CDK4/6i and fulvestrant.

### CDK-specific siRNA-mediated knockdown reveals CDK2 and CDK6 as key modulators driving resistance in letrozole-resistant ER+ BC cells

**Fig. 2:**
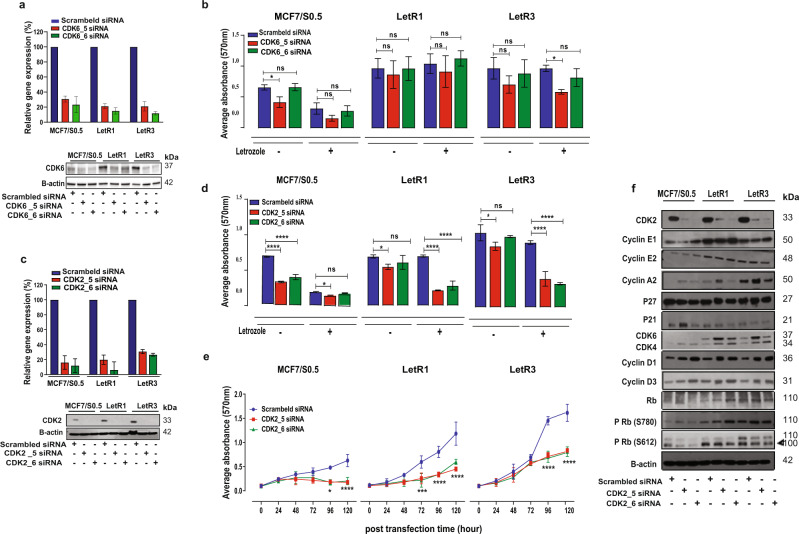
siRNA-mediated CDK2 knockdown re-sensitizes letrozole-resistant cells to letrozole. The effect of CDK2 or CDK6 silencing in letrozole-resistant (LetR) and parental MCF7/S0.5 cells transfected with two different CDK6-specific (CDK6_5 and CDK6_6), CDK2-specific (CDK2_5 and CDK2_6) or scrambled (control) siRNAs. **a** Quantitative RT-PCR and western blotting verifying reduction of CDK6 at mRNA (48 h) and protein levels (96 h) post-transfection with CDK6-specific siRNAs. The expression was normalized using *PUM1* gene, while β-actin was used as protein loading control. **b** Cell growth in the presence or absence of letrozole 96 h post-transfection with CDK6-specific siRNAs, as assessed by crystal violet assay. **c** Quantitative RT-PCR and western blotting verifying reduction of CDK2 at mRNA (48 h) and protein levels (96 h) post-transfection with CDK2-specific siRNAs. The expression was normalized using *PUM1* gene and β-actin was used as protein loading control. **d** Cell growth in the presence or absence of letrozole at 96 h post-transfection, as assessed by crystal violet assay. **e** Cell growth in the presence of letrozole at different time points following CDK2-specific siRNA transfection, as assessed by crystal violet assay. **f** Western blotting analysis of G1/S transition cooperative cascades at 96 h after CDK2-siRNA transfection in the absence of letrozole. β-actin was used as loading control. The cell growth is represented as average absorbance at 570 nm of triplicates mean ± SD. Statistically significant differences calculated by two-way ANOVA are shown as ns *p* > 0.05, *p ≤ 0.05, ***p ≤ 0.001, and ****p ≤ 0.0001.

Next, we evaluated the role of CDK6 and CDK2 in promoting resistance to AI letrozole using siRNAs specifically targeting CDK2 or CDK6 and a scrambled siRNA (control). Efficient silencing of CDK6 was obtained in CDK6-siRNA transfected cells (LetR and MCF7/S0.5) compared with the control siRNA, as determined by quantitative RT-PCR and western blotting (Fig. 2a and Supplementary Fig. 4a). However, CDK6 silencing did not affect the growth of LetR cells in the absence of letrozole and did not re-sensitize LetR cells to letrozole, as assessed by crystal violet assay (Fig. 2b). Efficient silencing of CDK2 was also obtained in CDK2-siRNA-transfected cells, as shown by quantitative RT-PCR and western blotting (Fig. 2c). Silencing of CDK2 re-sensitized LetR cells to letrozole upon exposure to letrozole (Fig. 2d), and a significant time-dependent growth inhibition was observed in CDK2-knockdown cells compared to their corresponding siRNA control in the presence of letrozole (Fig. 2e). In contrast, silencing CDK2 did not, or only marginally, reduce growth of LetR cells in the absence of letrozole, and did not induce significant alterations in the distribution of both LetR cells in the S-phase of the cell cycle (Fig. 2d and Supplementary Fig. 5a, b). These results indicate the importance of CDK2 in AI resistance and suggest that CDK2 role is E2-dependent. The role of CDK2 was further investigated by assessing the protein expression of the G1/S transition cooperative cascades after CDK2 siRNA-mediated knockdown in LetR cells. Western blotting analysis showed no consistent alteration in the expression of the CDK2 binding cyclins (E1, E2, and A2) or CDK inhibitors (p21^cip^ and p27^kip^) with the two CDK2-siRNAs in LetR1 and LetR3 cells, indicating that re-sensitization to letrozole was a result of direct inhibition of CDK2 (Fig. 2f and Supplementary Fig. 4b). We further examined the phosphorylation status of the Rb protein at two different CDK-dependent phosphorylation sites and found a slight decrease in Rb ser612 (CDK2-dependent) phosphorylation levels, and a marked increase in the phosphorylation levels of the basal activation site ser780 (CDK4/6- and CDK2-dependent). In association, a pronounced upregulation of D-type cyclins (D1 and D3), and CDK4 and CDK6 was identified. Notably, upregulation of these D-type cyclins and CDK4, but not CDK6, was also observed in the MCF7/S0.5 cells, and these alterations were not associated with hyperphosphorylation of Rb ser780 in MCF7/S0.5 cells. Thus, activation of the early G1/S transition cascade cyclin D-CDK4/6-Rb following CDK2 knockdown was derived by CDK6, which is suggested as a rescue mechanism in LetR cells, but not in the sensitive cells. Collectively, these results indicate that CDK2 is a potential therapeutic target for letrozole re-sensitization, while co-targeting CDK6 might be required to suppress a secondary mechanism of resistance in CDK6, p-CDK2 and/or cyclin E1 upregulated resistant ER+ BC cells.

### CDK2i synergize with CDK4/6i and ET to impair growth of AI-resistant and combined CDK4/6i and fulvestrant-resistant ER+ BC cells

**Fig. 3:**
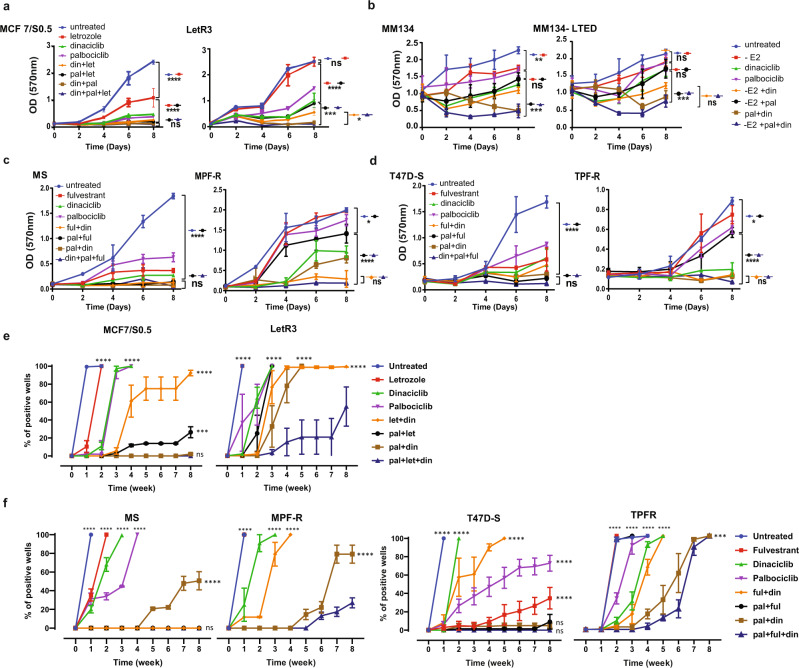
The triple combination with CDK2i, CDK4/6i and ET delays emergence of resistance in AI-resistant and combined CDK4/6i and fulvestrant-resistant ER+ BC cells. Resistant cells (LetR3, MM134-LTED, MPF-R, and TPF-R) and sensitive cells (MCF7/S0.5, MM134, MS, T47D-S, respectively) were used. **a**–**d** Cell growth assays were performed over 8 days of treatment with AI (letrozole (let) 1 μM; or absence of E2, 1 μg/ml), fulvestrant (ful, 100 nM), CDK2i dinaciclib (din, 10 nM), or CDK4/6i palbociclib (pal, 150 nM) alone or in different combinations, as assessed by crystal violet assay. The data represent the mean of three biological replicates ± SD. **e**, **f** Outgrowth of resistant colonies was evaluated following treatment with AI (letrozole (let), 1 μM; or absence of E2, 1 μg/ml), fulvestrant (ful, 100 nM), CDK2i dinaciclib (din, 5 nM), or CDK4/6i palbociclib (pal, 150 nM) alone or in different combinations over a period of 8 weeks. Wells showing ≥50% confluency were considered positive. Statistically significant differences calculated by one-way (**a**–**d**) or two-way (**e** and **f**) ANOVA are shown as ns *p* > 0.05, *p ≤ 0.05, **p ≤ 0.01, ***p ≤ 0.001, and ****p ≤ 0.0001.

Based on the data above, we examined whether AI-resistant cells that express high CDK6, p-CDK2, and/or cyclin E1 exhibited primary resistance to combined CDK4/6i (palbociclib) and AI, as well as whether adding CDK2 inhibitor (CDK2i, dinaciclib) to CDK4/6i and ET could overcome the resistance (Fig. 3a, b). As expected, we found that MM134-LTED were resistant to combined CDK4/6i and AI (absence of 1 μg/ml E2) and LetR3 cells exhibited increased growth after 6 days of treatment with combined CDK4/6i and letrozole, compared to the sensitive cell lines (MM134 and MCF7/S0.5, respectively). These suggest that AI-resistant cells exhibiting high expression of this combined biomarker are more susceptible to develop resistance to combined CDK4/6i and AI. Adding CDK2i to combined CDK4/6i and AI significantly inhibited the growth of both AI-resistant cell lines. The resistance to combined CDK4/6i and AI and efficacy of the triple combination in AI-resistant cells were also demonstrated by CellTiter-Blue, which measures the metabolic activity/viability of living cells (Supplementary Fig. 6a, b). Calculations of the combination index (CI) showed that CDK4/6i, AI and CDK2i exhibited synergistic activity when combined (Supplementary Fig. 6e and Supplementary Table 4).

We further evaluated whether addition of CDK2i to CDK4/6i and fulvestrant could inhibit cell growth of combined CDK4/6i and fulvestrant-resistant ER+ BC cells that exhibited high expression of CDK6, p-CDK2 and/or cyclin E1. Evaluation of the growth of MPF-R and TPF-R and their parental-sensitive MS and T47D-S cells showed that the addition of CDK2i to combined CDK4/6i and fulvestrant significantly inhibited cell growth of MPF-R and TPF-R cells (Fig. 3c, d). The efficacy of the triple combination was also confirmed by CellTiter-Blue (Supplementary Fig. 6c, d). Calculations of the combination index (CI) showed that CDK4/6i, fulvestrant and CDK2i exhibited synergistic activity when combined also in these cell lines (Supplementary Fig. 3f and Supplementary Table 3).

Our results highlight the importance of inhibiting both cyclin D-CDK4/6 and cyclin E-CDK2 cascades by targeted therapy in combination with ET. When comparing the inhibition obtained by combined CDK2i and ET with that of the triple combination containing CDK4/6i, more efficient inhibition of cell growth and viability was obtained by the triple combination, although this was not consistently significant in short-term culture experiments (Fig. 3a–d and Supplementary Fig. 6a–d).

In line with these results, we observed that the triple combination reduced the growth of resistant colonies in LetR3, MPF-R and TPF-R cells over the 8-week treatment, even when using low concentrations of CDK2i (Fig. 3e, f and Supplementary Fig. 7), while resistant clones appeared within 1–2 weeks when cells were exposed to the double combination of CDK4/6i or CDK2i with an ET (AI or fulvestrant). Thus, there is a substantially stronger efficacy of the triple combination over the other double combinations (CDK4/6i or CDK2i and ET) in long-term growth experiments, while both combined CDK4/6i and ET (AI or fulvestrant) and the triple combination (addition of CDK2i) prevented formation of resistant clones in the sensitive cell lines (MCF7/S0.5, MS, and T47D-S). Taken together, these results suggest that ER+ BC patients whose disease progressed on AI-monotherapy and with tumors expressing high CDK6, p-CDK2 and/or cyclin E1 may not benefit from the sequential treatment of CDK4/6i and ET, and that incorporation of CDK2i in this combination is required.

To assure that our findings with CDK2i dinaciclib were a result of CDK2-specific targeting, we tested another CDK2i, SNS-032, which also inhibits CDK7 and CDK9^36,37^, in combination with CDK4/6i palbociclib and ET letrozole (Supplementary Fig. 8a–d). The effect of SNS-032 on cell growth inhibition (LetR cells and the parental MCF7/S0.5 cell line) was comparable to dinaciclib, suggesting that the growth inhibition is mediated by on-target effects. Furthermore, we have tested the CDK2/4/6i PF-06873600 and the CDK2/9i fadraciclib in all our resistance models, including MCF7/LetR3, MS/MPF-R, T47D-S/TPF-R and MM134/LTED (Supplementary Fig. 9). The data show that triple combination with ET, CDK4/6i and either CDK2/4/6i or CDK2/9i efficiently inhibited growth of both sensitive and resistant cells in all cell models, which concurs with our findings with dinaciclib and SNS-032. Furthermore, CDK2/4/6i PF-06873600 combined with ET (letrozole/-E2 or fulvestrant) was as effective as the respective triple combination.

It has recently been reported that abemaciclib, another CDK4/6i, can inhibit CDK2 in addition to its primary target, CDK4/6^38^. Therefore, we examined whether combined abemaciclib and ET could exhibit similar cell growth inhibitory effects as the triple combination of CDK2i (dinaciclib) and CDK4/6i (palbociclib) and ET in LetR3, MPF-R and TPF-R cells (Supplementary Fig. 10a, b). The triple combination of CDK2i (dinaciclib), CDK4/6i (palbociclib) and ET (AI or fulvestrant) caused significantly more growth inhibition than combined CDK4/6i (abemaciclib) and ET in these resistant cells, while comparable inhibition was obtained with both combinations in the sensitive cells. These results indicate that CDK2 and CDK4/6 targeting induced by abemaciclib is less efficient than the triple combination in resistant ER+ BC cells expressing high CDK6, p-CDK2, and/or cyclin E1. Moreover, our data indicate that cells resistant to the CDK4/6i palbociclib show cross-resistance to another CDK4/6i (abemaciclib), as recently shown^39,40^.

### Triple combination with CDK2i, CDK4/6i, and ET suppresses proliferation and induces apoptosis in AI-resistant and combined CDK4/6i and fulvestrant-resistant ER+ BC cells

**Fig. 4:**
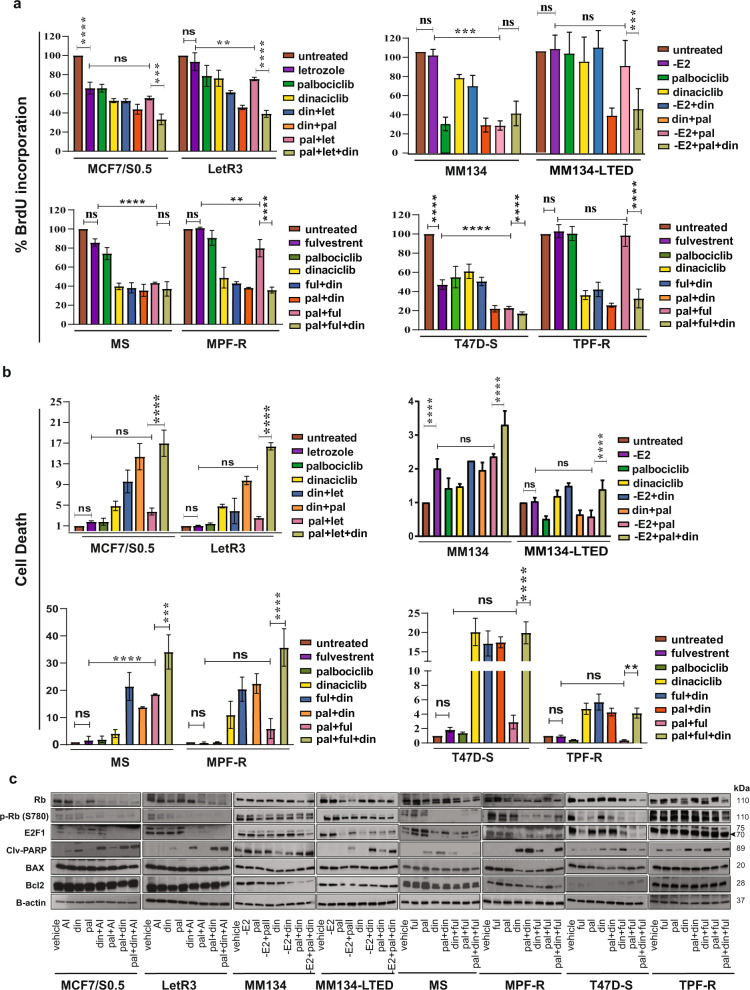
Triple combination of CDK2i, CDK4/6i and ET inhibits proliferation and induces apoptosis in AI- and combined palbociclib and fulvestrant-resistant ER+ BC cells. The effect on proliferation and apoptosis of AI (letrozole (let), 1 μM; or absence of E2, 1 μg/ml), fulvestrant (ful, 100 nM), CDK2i dinaciclib (din, 10 nM), or CDK4/6i palbociclib (pal, 150 nM) alone or in different combinations was assessed in the resistant cells (LetR3, MM134-LTED, MPF-R and TPF-R) and the parental cells (MCF7/S0.5, MM134, MS and T47D-S, respectively). Cells were treated for 24 h except for TPF-R and T47D-S, which were treated for 72 h due to their slower growth rate. **a** Inhibition of cell proliferation was evaluated by BrdU incorporation assay measuring newly synthesized DNA. **b** Apoptosis was determined by a Cell Death Detection ELISA kit measuring cytoplasmic histone-associated DNA fragments. The readout was normalized to cell numbers. **c** Western blotting analysis evaluating the level of cell cycle- and cell death-associated proteins. β-actin was used as loading control. The data represent the mean of three biological replicates ± SD. Statistically significant differences calculated by one-way ANOVA are shown as ns >0.05, *p ≤ 0.05, **p ≤ 0.01, ***p ≤ 0.001, and ****p ≤ 0.0001.

Next, we examined whether the growth inhibition obtained by the triple combination was caused by reduced cell proliferation and/or induced apoptosis. We performed a BrdU incorporation assay to measure proliferation (Fig. 4a) and a DNA fragmentation assay to evaluate apoptosis (Fig. 4b) after exposure to different drug combinations in AI- and combined CDK4/6i and fulvestrant-resistant cells. We observed that the triple combination caused a significant decrease in cell proliferation and substantially induced apoptosis in all tested ER+-resistant BC cells (Fig. 4a, b). Combined CDK4/6i (palbociclib) and ET induced only a slight inhibition of proliferation in the resistant cells (LetR3, MM134-LTED, MPF-R, and TPF-R) compared with the sensitive cells (MCF7/S0.5, MM134, MS, and T47D-S). Importantly, the addition of CDK2i (dinaciclib) to this combination significantly synergized the antiproliferative effect in the resistant cells. Furthermore, inhibition of cell proliferation by the triple combination was also confirmed by cell cycle analysis. Cells under aromatase-dependent growth conditions (LetR3 and MCF7/S0.5) were found to be arrested in the G0/G1 phase after treatment with the triple combination (Supplementary Fig. 11a, b). This arrest was not correlated with cellular senescence in LetR3 cells, while sensitive MCF7/S0.5 cells showed a significant increase of senescence-associated β-galactosidase activity (Supplementary Fig. 11c, d). In contrast, cell arrest in the G2/M phase was obtained after treatment with the triple combination in combined palbociclib and fulvestrant-resistant cells (MPF-R and TPF-R) and parental cells (MS and T47D-S, respectively) (Supplementary Fig. 12a, b), which is in line with previous studies that addressed the effect of CDK2i on the cell cycle^41–44^.

Furthermore, apoptosis analysis revealed that combined CDK4/6i and ET could not induce cell death in resistant cells (Fig. 4b). However, addition of CDK2i to combined CDK4/6i and ET significantly promoted apoptosis, which was also reflected at the protein level by increased cleaved PARP levels (Fig. 4c). Overall, our data suggest that the synergistic effect induced by the triple combination on cellular growth, viability and apoptosis (Fig. 3; Supplementary Figs. 6a, b and 4) was mediated by both cytostatic and cytotoxic activity.

### Triple inhibition of CDK2, CDK4/6, and ER modulates c-Myc and S6 in AI-resistant and combined CDK4/6i and fulvestrant-resistant ER+ BC cells

**Fig. 5:**
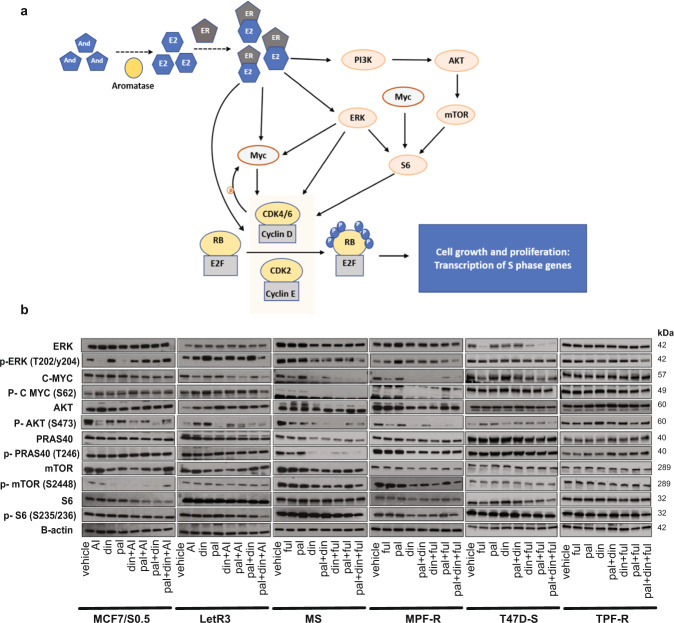
Triple combination of CDK2i, CDK4/6i, and ET inhibits S6 kinase axis in ER+/HER2- BC models. **a** Scheme showing simplified interactions between oncogenic pathways upstream of G1/S-phase transition, including PI3K/AKT/mTOR, RAS/MEK/ERK, and c-Myc. **b** Western blotting analysis of key proteins of these oncogenic pathways in the resistant (LetR3, MPF-R, and TPF-R) and sensitive cells (MCF7/S0.5, MS, and T47D-S, respectively) treated with letrozole (let, 1 μM), fulvestrant (ful, 100 nM), dinaciclib (din, 10 nM), or palbociclib (pal, 150 nM) alone or in different combinations. Cells were treated for 24 h, except for TPF-R and T47D-S, which were treated for 72 h due to their slower growth rate. β-actin was used as loading control.

Several studies have reported cross-talk and activation of G0/G1 upstream mitogenic pathways, including c-Myc, RAS/MEK/ERK and PI3K/AKT/mTOR, that promotes cell survival, proliferation and resistance to treatment, such as CDK4/6i^39,40,45–49^ (Fig. 5a). Based thereon, we further identified the underlying mechanisms of synergism obtained by the triple combination of CDK2i, CDK4/6i and ET by evaluating the activity of these mitogenic pathways using western blotting. Notably, pronounced inhibition of phosphorylated ribosomal protein S6 (p-S6), a common downstream mediator of these mitogenic pathways^48–50^, was observed with the triple combination in all resistant cells (LetR3, MPF-R, and TPF-R) (Fig. 5b and Supplementary Fig. 9). This suggests a possible inhibition of the upstream oncogenic pathways leading to inhibition of the S6 kinase axis. Therefore, the active status of c-Myc, and the RAS/MEK/ERK and PI3K/AKT/mTOR pathways were assessed (Fig. 5b and Supplementary Fig. 13). Interestingly, a reduction of phosphorylated ERK (p-ERK), indicative of reduced RAS/MEK/ERK pathway activity, and a reduction of the phosphorylated form of c-Myc ser62 (p-c-Myc), indicative of reduced estrogen signaling, was consistently obtained for the triple combination compared to the combined CDK4/6i and ET in the resistant cells (LetR3, MPF-R, and TPF-R). Moreover, we did not observe a consistent reduction in PI3K/AKT/mTOR pathway as evaluated by the phosphorylated level of AKT, and the two AKT downstream regulators PRAS40 and mTOR.

It is well-known that phosphorylation of c-Myc at ser62 induced by ERK and the cell cycle regulators CDK4/6 and CDK2 leads to further stabilization and transcriptional activation^40,51–54^. Consequently, the inhibitory effect on cell growth induced by CDK2i and CDK4/6i combined with ET could be, at least in part, a result of the impaired oncogenic activity of c-Myc that further inhibits S6 in AI-resistant and combined CDK4/6i and fulvestrant-resistant ER+ BC cells.

### Triple combination of CDK2i, CDK4/6i, and AI suppresses growth of AI-resistant autocrine tumor xenografts

**Fig. 6:**
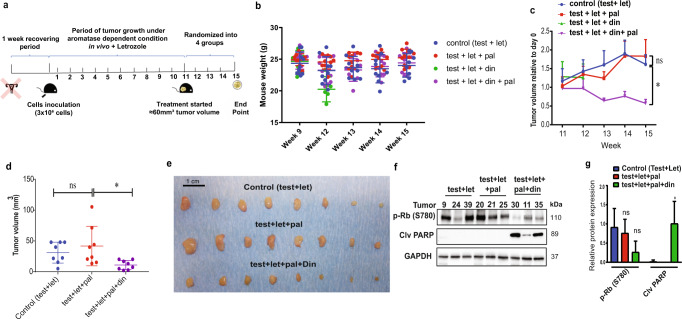
Combined treatment with CDK2i, CDK4/6i, and AI suppresses autocrine estrogen-dependent growth of BC tumors in a postmenopausal xenograft model. **a** Scheme by week showing the protocol of the in vivo model simulating postmenopausal BC patients with autocrine estrogen-dependent tumors. **b** Mouse bodyweights were measured at week 9 and then weekly from week 12. **c**, **d** Orthotopic LetR3 tumors grown in the presence of 250 μg testosterone (test) + 10 μg letrozole (let) were randomized to 4 groups (*N* = 8 per group); control (250 μg test + 10 μg let), 12.5 mg/Kg CDK4/6i palbociclib (pal), 10 mg/Kg CDK2i dinaciclib (din), 12.5 mg/Kg pal + 10 mg/Kg din. Test + Let were administered to all treatment groups by subcutaneous injection in 0.5% carboxymethylcellulose (CMC), palbociclib was administered by oral gavage in 25% HP-beta-cyclodextrin (HPC), and dinaciclib was administered intraperitoneally (IP) in 5% HPC. All drugs were administered daily for 5 weeks. Mice from test + let + din group were sacrificed after 1 week of treatment due to toxicity. **c** Tumor growth curve relative to the treatment starting point (week 11). **d** Tumor volume at the endpoint of treatment (week 15). Data are shown as mean tumor volume ± SEM and the *p*-values are calculated using one-way ANOVA test at the endpoint of the treatment **e** Picture of the resected tumors. **f** Western blotting analysis of cell cycle arrest and cell death markers in lysates from three tumors of each treatment group. GAPDH was used as loading control. **g** Densitometry analysis of western blotting bands from three different tumors of each group was performed using ImageJ software and normalized to GAPDH. Data are shown as mean area under the curve (AUC) normalized to the loading control ± SD. Statistically significant differences calculated by one-way ANOVA are shown as ns >0.05, *p ≤ 0.05.

Next, we evaluated whether addition of CDK2i (dinaciclib) to the combined CDK4/6i (palbociclib) and AI (letrozole) standard treatment efficiently abrogated growth of letrozole-resistant MCF7-derived BC tumors in vivo. First, we developed a new xenograft model simulating the postmenopausal condition wherein breast tumors grow by aromatizing non-ovarian androgens without genetic engineering. In this model, immune-compromised NOG CIEA mice were ovariectomized and supplemented with testosterone, which can be converted by aromatase to estradiol and promotes cancer cell growth in the inoculated cancer cells. In this setting, injection of 3 × 10^6^ cells into the mammary fat pad of mice resulted in tumor formation for both parental MCF7/S0.5 and corresponding AI-resistant LetR3 cells. However, only LetR3 tumors were able to grow by an autocrine source of estrogen (Supplementary Fig. 14a–c), indicating that local production of estrogen by intra-tumoral aromatase was sufficient to stimulate tumor growth of AI-resistant cells. Evaluation of the efficacy of the triple combination on this model was performed using a lower dosage of CDK2i than reported in other studies^41,44,55^, emphasizing the need to optimize the utility of lower doses clinically. After tumors reached the size of 60 mm^3^ (11 weeks after orthotopic inoculation of cells in the presence of testosterone 250 μg+ letrozole 10 μg/day in 0.5% carboxymethylcellulose by subcutaneous administration), mice were randomized into treatment groups with palbociclib alone (12.5 mg/Kg in 25% HP-beta-cyclodextrin by oral administration), dinaciclib alone (10 mg/kg in 5% HP-beta-cyclodextrin by intraperitoneal administration (IP)) and the combination of palbociclib and dinaciclib, or vehicle (25% and 5% HP-beta-cyclodextrin orally and IP, respectively) for 5 weeks (Fig. 6a). All mice received daily supplementation with testosterone + letrozole after initiating treatment. Notably, the combination of letrozole and dinaciclib was toxic to the mice after several administrations, and thus the mice in this treatment group were sacrificed earlier than the mice in the other groups (Fig. 6b). However, in line with our in vitro analysis, tumors of mice treated with combined palbociclib and letrozole progressed at the same rate as those treated with letrozole alone (Fig. 6c–e). In contrast, tumors of mice treated with the triple combination of dinaciclib, palbociclib and letrozole were significantly smaller compared to the combined palbociclib and letrozole treated group (*p* = 0.0256) (Fig. 6c–e). No significant outliers were identified by Grubbs’ method. Importantly, western blotting analysis of the xenograft tumors treated with the triple combination showed lower levels of p-Rb S780 and substantially higher levels of cleaved PARP (Fig. 6f, g), supporting the anti-tumor activity reported in vitro (Fig. 4). Expression levels of CDK6, p-CDK2, and cyclin E1 were also evaluated in xenograft tumors of different treatment groups by western blotting (Supplementary Fig. 14d, e).

### High expression of CDK6, p-CDK2, and/or cyclin E1 correlates with shorter PFS in ER+ ABC patients treated with AI-monotherapy or combined CDK4/6i and ET

**Fig. 7:**
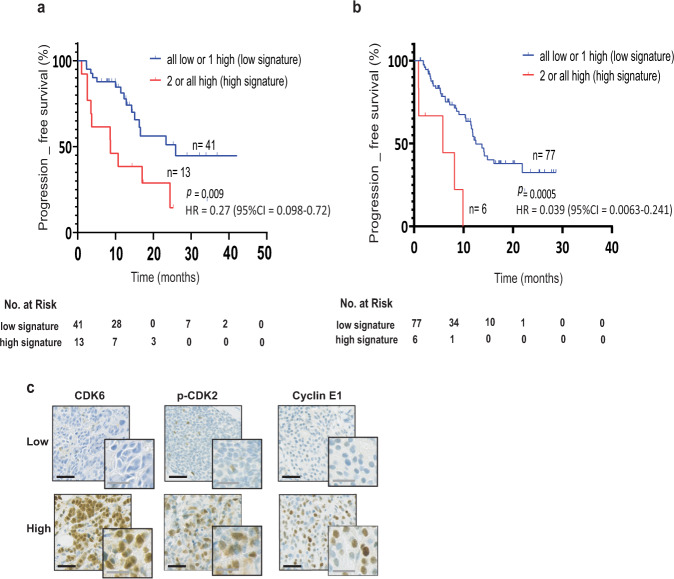
High expression of CDK6, p-CDK2, and/or cyclin E1 is associated with low progression-free survival in ER+ ABC patients treated with AI-monotherapy or combined CDK4/6i and ET. Kaplan–Meier plots evaluating PFS according to a combined score based on the levels of three biomarkers CDK6, p-CDK2, and cyclin E1, in ER+ metastatic lesions from BC patients in the advanced setting. **a** Cohort of patients receiving AI-monotherapy. **b** Cohort of patients treated with combined CDK4/6i and ET. The cutoff values used were: CDK6 *H*-score > 0, p-CDK2 *H*-score ≥ 75, and cyclin E1 *H*-score ≥ 100. A two-sided *p*-value calculated using log-rank testing is shown. **c** Representative micrographs of immunohistochemistry analysis of all breast cancer metastasis sections showing low or high CDK6, p-CDK2 and cyclin E1 expression (black scale bar: 100 µm, gray scale bar: 50 µm).

Finally, we evaluated the clinical relevance of the three proteins CDK6, p-CDK2 and cyclin E1, found to be upregulated in our AI-resistant and combined CDK4/6 and fulvestrant-resistant BC cells. As CDK6 and cyclin E1 have been reported as poor predictive biomarkers in retrospective analyses of clinical trials evaluating the combination of ET and CDK4/6i^34,56^, we suggested a combined score based on the levels of CDK6, p-CDK2, and cyclin E1 proteins to power the significance of our analysis. Thus, we evaluated the clinical relevance of the combined score of these three proteins in BC samples of a cohort of patients receiving AI-monotherapy (*N* = 54) and in a cohort of patients treated with combined CDK4/6i and ET (*N* = 83), both in the advanced setting. Expression levels of CDK6, p-CDK2 and cyclin E1 were evaluated in full sections of metastatic lesions of ER+ BC patients by immunohistochemistry. Initially, each protein was analyzed separately and the cutoff value was determined based on the median *H*-score and survival significance in Kaplan–Meier curve for the AI-treated cohort (CDK6 *H*-score > 0, *p* = 0.84; p-CDK2 *H*-score ≥ 75, *p* = 0.9; and cyclin E1 *H*-score ≥ 100, *p* = 0.02; Supplementary Fig. 15a–c). The same cutoff values were also used for the combined CDK4/6i and ET-treated patients (CDK6 *p* = 0.001; p-CDK2 *p* = 0.127; and cyclin E1 *p* < 0.0001; Supplementary Fig. 15d–f). For AI-treated patients, although only the level of cyclin E1 showed a significant correlation with PFS, combining the biomarkers increased the power of that significance (*p* = 0.009) (Fig. 7a). For the cohort of patients treated with combined CDK4/6i and ET, the level of CDK6 showed a significant correlation with PFS, and combining the biomarkers showed comparable significance (*p* = 0.0005) (Fig. 7b). In these Kaplan–Meier curves (Fig. 7a, b), patients were stratified into high (high expression of at least two of the three biomarkers) and low (none or only one of the biomarkers exhibiting high expression) signatures. Representative immunohistochemistry stainings of low and high CDK6, p-CDK2 and cyclin E1 levels are shown (Fig. 7c). For the AI-monotherapy cohort, the median time to progression was 8.6 months for the patients with tumors exhibiting the high signature compared to 25.9 months for those with the low signature (Fig. 7a). For the combined CDK4/6i and ET-treated cohort, the median time to progression was 5.8 months for patients with tumors exhibiting the high signature compared to 12.47 months for those with the low signature.

The *χ*^2^ and Fisher’s exact tests did not identify statistically significant differences between high- and low-signature groups of the combined biomarkers and clinicopathological parameters of the primary tumors of AI-treated patients or combined CDK4/6 and ET-treated patients (Supplementary Table 5). Cox proportional hazard regression analysis of PFS according to the signatures of CDK6, p-CDK2 and cyclin E1 and clinicopathological parameters of the metastatic disease showed that the combined biomarkers signature was an independent prognostic factor of PFS in both tested cohorts (Supplementary Table 6). Although endocrine status and lines of therapy also showed a significant correlation with PFS in univariate Cox’s proportional hazards regression analysis in the cohort of combined CDK4/6 and ET-treated patients, multivariate analysis showed that only the signature of the combined biomarkers significantly correlated with PFS (Supplementary Table 6).

Overall, our data suggest that metastatic ER+ patients treated with AI-monotherapy or combined CDK4/6i and ET whose tumors exhibit high levels of CDK6, p-CDK2 and/or cyclin E1 are associated with worse clinical outcome and may benefit from the addition of a CDK2i in their treatment strategy.

## Discussion

Adjuvant AI treatment of ER+ BC is highly effective, but ~25% of patients experience recurrence. For these patients, combined treatment with CDK4/6i and ET as first-line treatment has demonstrated significant improvement of PFS and OS^15–21^. Nonetheless, primary resistance is observed in some patients, and patients who initially respond will inevitably develop resistance, emphasizing the need to identify biomarkers of resistance for better treatment selection. Understanding the heterogenous resistance mechanisms to AI-monotherapy may provide important information and lead to the development of novel combined treatments in the advanced setting. Herein, we identified high levels of CDK6, p-CDK2 and cyclin E1 to be associated with resistance to both AI-monotherapy and combined CDK4/6i and ET. Using these three key molecules as a combined biomarker, we were able to show that the combined score was an independent prognostic factor that could identify a subgroup of ER+/HER2– ABC patients with poor outcome on the standard treatments and that may benefit from a triple combination therapy including CDK4/6i, CDK2i, and ET.

In postmenopausal BC patients, the estrogen supply may originate from both circulatory estrogen uptake and locally synthesis in tumor cells and surrounding adipocytes and fibroblasts, however the most important estrogen source driving AI resistance remains debatable^35,57,58^. Considering the complicated regulation of aromatase enzyme and the high aromatase expression found in BC cells^59–61^, some preclinical studies have used models that mimic local synthesis by stably transfecting BC cells with aromatase cDNA^26,62–64^. Although overexpression of the enzyme has been reported in BC cells, their levels remained low, and a weak correlation between aromatase levels and clinical outcome has been reported^65–67^. Thus, most studies moved towards BC cell models of AI resistance that mimic systemically delivered estrogen by long-term estrogen deprivation (LTED)^68,69^. Since the differences in the identified AI-resistance mechanisms are likely due to the varying sources of estrogen, we initiated our investigation by evaluating AI-resistant ER+ BC cell models mimicking both types of estrogen supply. The first model represents resistance to the AI letrozole, wherein BC cells growth depends on endogenous aromatase enzyme^35^ (LetR), while the second model is based on the LTED BC cell model (MM134-LTED). Noteworthy, a specific AI-resistance mechanism in LetR cells has not been identified by the group who developed the model^35^ and the suggested mechanisms of ligand-independent activation is not supported by our in vitro and in vivo data.

In both our AI-resistant ER+ BC cell models, we observed high levels of CDK6, p-CDK2 and/or cyclin E1, which are regulators of G1/S-phase transition of the cell cycle, in the resistant cells, indicating that the role of the G1/S cooperative cascades in AI-resistance mechanisms is independent of the source of estrogen. Importantly, we observed similar alterations in two cell models resistant to combined treatment of CDK4/6i and fulvestrant.

Interestingly, we found that siRNA-mediated silencing of CDK6 in the AI letrozole-resistant cells (LetR1 and LetR3) had no distinct impact on the response to letrozole. In contrast, the siRNA-mediated inhibition of CDK2 restored the sensitivity of these cells to letrozole, consistent with other studies that reported restoration of sensitivity to letrozole by silencing CDK2 in genetically engineered (LMW-E/aromatase induced) cells, suggesting CDK2 as a potential therapeutic target^26,70^. Importantly, we observed activation of the cyclin D-CDK4/6-Rb cascade following silencing of CDK2, suggesting that cyclin D-CDK4/6 may not be the primary mechanism driving AI resistance, but may compensate for the inhibition of cyclin E-CDK2 and promote cell cycle re-entry in the resistant cells. Interestingly, other studies have suggested an alternative regulation of G1/S-transition wherein cyclin E-CDK2 is activated and compensates for cyclin D-CDK4/6 inhibition^71,72^.

Based on the siRNA findings, we evaluated the therapeutic efficacy of dinaciclib, a pan CDK inhibitor that exhibits the highest affinity for CDK1/2, in combination with the CDK4/6i palbociclib and ET in the AI-resistant BC cell models. We observed that combined palbociclib and AI treatment had a limited growth inhibitory effect on these AI-resistant cells and resistant clones developed quickly in outgrowth assays. However, adding dinaciclib to combined palbociclib and AI durably inhibited cell growth and delayed acquisition of resistance in these cells. Importantly, triple combination of dinaciclib, palbociclib and the ET fulvestrant also significantly inhibited growth in combined CDK4/6i and fulvestrant-resistant BC cells. In line with these in vitro data, our letrozole-resistant autocrine in vivo model exhibited resistance to combined palbociclib and letrozole, while the triple combination incorporating dinaciclib efficiently suppressed tumor growth.

Toxicity must be considered when combining the three drugs and frequently leads to treatment discontinuation or dose reduction. Toxicity associated with high doses of dinaciclib (20 and 50 mg/m^2^) as monotherapy has been reported in advanced BC clinical trials, particularly in triple-negative BC (TNBC)^73,74^. Therefore, we tested a lower concentration of dinaciclib in the outgrowth (5 nM) and in the xenografts model (10 mg/kg)^41,44^. Furthermore, our data show that combined CDK2/4/6i PF-06873600 and ET is as effective as the triple combination with ET, CDK4/6i and either dinaciclib, CDK2/4/6i PF-06873600 or CDK2/9i fadraciclib, suggesting that this double combination could be an alternative therapeutic strategy. These findings concur with a recent publication that showed that palbociclib-resistant cells exhibiting induction of MYC oncogene and cyclin E/CDK2 activity were sensitive to the CDK2/4/6i PF-06873600^75^. However, our study evaluated the efficacy of PF-06873600 and the role of high p-CDK2, cyclin E1 and CDK6 not only in CDK4/6i and ET-resistant, but also in AI-resistant cell models and xenografts.

Although the efficacy of the single agent dinaciclib in arresting cell cycle and inducing cell death has been previously reported in different types of cancer^41,43,76^, the durable growth inhibition induced by dinaciclib, as single agent or in double and triple drug combinations, has not been studied in ER+ BC. We found that triple combination with CDK2i, CDK4/6i and ET is synergistic and exhibited both cytostatic and cytotoxic effects. Our analysis suggests that the growth inhibition induced by the triple combination may be caused by suppression of c-Myc phosphorylation at Ser62. This is in line with a recent study that reported downregulation of phosphorylated c-Myc following CDK2 silencing as a mechanism to restore sensitivity in a BC cell line model resistant to CDK4/6i single agent^40^. Thus, our findings provide strong evidence that co-targeting CDK2 and CDK4/6 in combination with ET can be utilized to overcome resistance to combined CDK4/6i and ET. However, the question remains whether AI or fulvestrant is the preferred ET backbone in this combined treatment. The triple combination of CDK2i dinaciclib, CDK4/6i palbociclib and fulvestrant was not directly tested on AI-resistant BC cells, although substitution to fulvestrant upon AI progression has proven to be beneficial^77^.

Although our study demonstrates that incorporation of dinaciclib synergizes with palbociclib and fulvestrant to overcome resistance in palbociclib and fulvestrant-resistant BC cells exhibiting high levels of CDK6, p-CDK2 and/or cyclin E1, it remains questionable whether the CDK4/6i should be maintained in the triple combination or switched to another targeted agent that can potentially inhibit the cyclin D-CDK4/6i-Rb cascade more efficiently (i.e., PI3K/mTOR/AKT targeted therapy).

Recent preclinical studies have suggested several single biomarkers of resistance to CDK4/6i, including Rb loss-of-function^32^, loss of FAT1^78^, *PIK3CA* mutations^25^, *ESR1* mutations^23^, overexpression or amplification of *CDK6*^79^ and *CCNE1*^34^, and alterations in FGFR^80^. These biomarkers, except Rb and FAT1 loss, have been retrospectively suggested to be associated with CDK4/6i and ET in clinical trials^23,34^. However, none of these markers is currently approved for clinical use, and identification of a predictive biomarker of response/resistance to the combined CDK4/6i and ET has proven challenging. Herein, we evaluated the levels of CDK6, p-CDK2, and cyclin E1 as a combined biomarker and found that high levels of at least two of these three markers correlated with poor clinical outcome in ER+/HER2- ABC patients treated with first-line AI-monotherapy. Interestingly, we found similar results in a cohort of ER+/HER2- ABC patients treated with combined CDK4/6i and ET. Thus, we propose that immunohistochemical evaluation of CDK6, p-CDK2 and cyclin E1 can be used clinically to identify patients who could experience early progression upon AI-monotherapy and likely would not significantly benefit from standard combined CDK4/6i and ET, but should receive triple combined therapy with a CDK2i to improve their survival. The predictive utility of this combined biomarker should be further evaluated in samples from larger randomized trials. Finally, biomarker-driven clinical trials evaluating dinaciclib and palbociclib combined with ET in ER+/HER2- postmenopausal ABC patients who progressed on AI, and also in patients who progressed on combined palbociclib and fulvestrant, eligible by evaluation of the expression of the three biomarkers in baseline biopsy specimens, may ultimately determine the utility of the combined biomarker.

## Methods

### Cell lines and standard culture conditions

The original MCF7 and T47D cell lines were obtained from the Breast Cancer Task Force Cell Culture Bank, Mason Research Institute and the original MDA-MP-134VI cell line was obtained from American Type Culture Collection (ATCC). MCF7 cells were gradually adapted to grow in low serum concentration to generate the MCF7/S0.5 subline used to establish the two AI (letrozole)-resistant cell lines (LetR1 and LetR3) by long-term exposure to 1 μM letrozole, as previously described^35^. MCF7/S0.5 cells were maintained in phenol red-free Dulbecco’s Modified Eagle’s Medium (DMEM)/F-12 (Gibco, Life Technologies) supplemented with 1% heat-inactivated fetal bovine serum (FBS; Life Technologies), 2 mM GlutaMAX™-1 (Life Technologies) and 6 ng/ml insulin (Sigma-Aldrich). LetR1 and LetR3 cells were cultured in a similar medium but were supplemented with 10% NCS (Sigma-Aldrich) in addition to 100 nM testosterone and 1 μM Letrozole. The long-term estrogen-deprived model MM134-LTED was used as a second AI-resistant cell model and was developed from the cell line MDA-MP-134VI that was routinely maintained in 1:1 DMEM (High Glucose, Life Technologies), L-15 (Life Technologies), and 10% FBS. The LTED-derived cell line was generated as recently described^81^. Briefly, MDA-MP-134VI cells were hormone-deprived and then maintained in improved minimal essential medium (IMEM, Life Technologies), 10% charcoal-stripped FBS (CSS, Life Technologies) for 6–12 months to acquire endocrine resistance. Before performing any experiments with AI-resistant models, all cell lines were cultured in 10% NCS contained DMEM/F12 medium (MCF7/S0.5 and LetR cells) or 10% CSS contained IMEM medium (MDA-MB-134 and MM134-LTED cells) to ensure that all cells were only influenced by the hormones supplemented during the experiment.

The combined palbociclib and fulvestrant-resistant BC models MPF-R and TPF-R were generated from the MCF7/S0.5 and T47D cell lines, respectively. Briefly, MPF-R and TPF-R were developed from the MCF7- and T47D-derived fulvestrant-resistant cells by long-term (3 to 4 months) incubation with combined 100 nM fulvestrant and 150 nM CDK4/6i palbociclib. The sensitive cell lines that were grown in parallel were termed MS and T47D-S, respectively. The MS and MPF-R were maintained in a similar medium as MCF7/S0.5 supplemented with 100 nM fulvestrant and 150 nM CDK4/6i palbociclib for MPF-R cells. T47D-S and TPF-R cells were maintained in RPMI 1640 medium (Gibco, Life Technologies) without phenol red supplemented with 1% glutamine, 5% FBS and 8 µg/ml insulin in addition to 100 nM fulvestrant and 150 nM CDK4/6i palbociclib for TPF-R. All cells were maintained at 37 °C in humidified air with 5% CO_2_, washed with phosphate-buffered saline (PBS, Sigma-Aldrich) and trypsinized (Sigma-Aldrich) when 70–80% confluence was attained. All cell lines underwent DNA authentication using Cell ID™ System (Promega) and mycoplasma testing (Lonza) before use in the described experiments.

### Specific chemicals and inhibitors

β-estradiol (E2), fulvestrant (ICI182;780; Tocris) and testosterone (Sigma-Aldrich) were dissolved in ethanol. CDK2is dinaciclib (SCH727965; S2768, Selleckchem), SNS-032 (S1145, Selleckchem), PF-06873600 (A16875, Adooq) and fadraciclib (HY-101212, Medchemexpress), AI letrozole (CGS 20267; S1235, Selleckchem) and enzalutamide (MDV3100, S1250, Selleckchem) were dissolved in dimethyl-sulfoxide (DMSO, Sigma-Aldrich). CDK4/6i palbociclib (PD332991; PZ0383, Sigma-Aldrich) was dissolved in double-distilled water (ddH_2_O).

### Global gene expression and microarray analysis

Global gene expression analysis was performed on RNA purified from the parental cell line MCF7/S0.5 and the 2 derived letrozole-resistant cell lines LetR1 and LetR3 (biological replicates: 6 LetR1, 3 LetR3 vs 6 MCF7/S0.5) using Affymetrix Gene Chip Human Genome U133 plus 2.0 (Affymetrix)^82^. Cells were grown in aromatase-dependent conditions without exposure to letrozole for 4 days (reaching 70% confluency). As a control, global gene expression analysis was also performed on RNA purified from MCF7/S0.5 and LetR1 grown in the presence of letrozole for a similar period. Total RNA was purified using RNeasy mini kit (Qiagen) according to the manufacturer’s instructions. Data were analyzed using Partek Genomic Suite (Partek Inc.). Raw Affymetrix intensity measurements were normalized and summarized into gene expression measurements using Robust Multiarray Average. Genes from the data set that exhibited twofold or greater alteration in expression with a false discovery rate (FDR) < 0.01 cutoff and one-way ANOVA *p* < 0.01 were considered significantly regulated. The list of significantly altered genes was subject to further pathway analysis using Ingenuity Pathways Analysis (IPA) (Qiagen) to evaluate whether these genes were part of functionally integrated biological networks. Raw microarray data have been deposited in the gene expression omnibus (GEO) database (GSE74391).

### RNA purification and quantitative real time PCR (RT-qPCR)

TRIzol (Life Technologies) was used for total RNA extraction and cDNA synthesis was performed using a RevertAid Premium Reverse Transcriptase Kit (Fermentas). Relative quantification of gene expression was performed using SYBR Green PCR Mastermix (Applied Biosystems) according to the manufacturer´s instructions. The primers were purchased from Qiagen: QT00019985 (transcript ID ENST00000265734, amplicon length 82) for *CDK6*, QT00005586 (transcript ID ENST00000266970, amplicon length 150) for *CDK2* and QT00041986 (transcript ID ENST00000262643, amplicon length 181) for *CCNE1*. The primer QT00029421 (transcript ID ENST00000257075, amplicon length 73) for *PUM1* was used as a reference gene. RT-qPCR reactions were performed on a StepOnePlus system (Applied Biosystems) and data were obtained from StepOne Software. All reactions were conducted in triplicates and the data were analyzed using the delta-delta CT method^83^.

### DNA extraction and copy-number variation assay

DNA was extracted using the DNeasy Blood and Tissue Kit (Qiagen) according to the manufacturer´s instructions. DNA concentration and purity were measured using NanoDrop spectrophotometer (ThermoFisher Scientific). The Taqman copy-number variant primers used were: Hs01548017_cn (location Chr.7:92606172 on GRCh38, amplicon length 101) for *CDK6*, Hs01055552_cn (location Chr.12:55966993 on GRCh38, amplicon length 111) for *CDK2*, and Hs07158517_cn (location Chr.19:29819809 on GRCh38, amplicon length 91) for *CCNE1* (encodes cyclin E1) (ThermoFisher Scientific). TaqMan Copy Number Reference Assay RNase P (4403326, location chr5:1253257 on GRCh38, amplicon length 88) from ThermoFisher Scientific was used as the internal control, according to the manufacturer’s protocol. StepOnePlus system was used for the RT-qPCR reactions and raw data were analyzed using CopyCaller software (Applied Biosystems).

### Knockdown by siRNA transfection

Chemical transfection with the lipofectamine 3000 transfection reagent (15282465, ThermoFisher Scientific) was used for siRNA-mediated knockdown according to the manufacturer’s instructions. For CDK6 and CDK2 knockdown experiments, 3 different siRNA were used for each; a non-targeting scrambled (control) siRNA (Sigma-Aldrich) and 2 different CDK-targeting siRNA; CDK6_5 (SI00605052, AAGACTCAAGGTGGTCAGTAA), CDK6_6 (SI00605059, TCTGAAGTGTTTGACATTTAA), CDK2_5 (SI00299775, AGGTGGTGGCGCTTAAGAAAA) and CDK2_6 (SI00299782, GACGGAGCTTGTTATCGCAAA) (Qiagen). Briefly, BC cells were plated at 0.125 × 10^5^ cell/well in 24 multi-well plates and incubated at 37 °C for 24 h. Subsequently, cells were transfected with 12.5 nM of the indicated siRNAs and the effect of the knockdown was evaluated in the presence and absence of AI letrozole at the time point indicated in the figure legends.

### Short-term cell growth and viability

BC cell lines were plated at 2000–3000 cell/well in 96 multi-well plates and incubated at 37 °C for 24 h before drug(s) (alone or in combinations) or vehicle were added for 6–8 days. Media were replenished every 3 days. Evaluation of cell growth was performed by crystal violet assay^84^ and the optical density (OD) was measured at 570 nm using Paradigm reader (Beckman Coulter). Cell viability was evaluated using CellTiter-Blue (G8080, Promega) according to the manufacturer´s instructions and fluorescence was measured at 560/590 nm using Paradigm reader.

### Colony outgrowth assay

Colony outgrowth assay was performed as previously described^85^. In brief, BC cells were plated at 750–1000 cells/well in 96-well plates and incubated at 37 °C for 24 h before treatment with vehicle or drug(s) (alone or in combinations). For each treatment condition, 48 wells were used as technical replicates. Medium was replenished every 5 days for 8 weeks. Positive wells were scored as ≥ 50% confluency and assessed weekly.

### BrdU (Bromodeoxyuridine) cell proliferation assay, cell death assay, and senescence-β Galactosidase assay

Cell proliferation was assessed using a BrdU cell proliferation kit (6813, Cell Signaling Technology), apoptosis was assessed using the Cell Death Detection ELISAPlus kit (11774425001, Roche), and senescence was assessed using a senescence-β Galactosidase (SA-β gal) staining kit (9860, Cell Signaling Technology) according to the manufacturer’s instructions. Briefly, BC cells were plated at 3000 cell/well in 96 multi-well plates for BrdU and death assays, and 0.125 × 10^5^ cells/well in 24 multi-well plates for senescence assay. Cells were then incubated at 37 °C for 24 h before treatment with the vehicle or drug(s) (alone or in combinations). OD for BrdU incorporation of the S-phase and the level of the nucleosome detected by the immunoreaction were measured at 450 nm and 405/490 nm, respectively, using Paradigm reader. For senescence, cells were photographed on an Olympus IX73 microscope and quantified by counting 200 cells in three different fields for each replicate.

### Cell cycle analysis by flow cytometry

BC cells (1 × 10^6^ cells /T75 tissue culture flask) were incubated at 37 °C for 24 h and either chemically transfected with specific siRNA at indicated concentrations or treated with the different drugs. Cells were then trypsinized and fixed in ice-cold 70% ethanol. Five hours post fixation, cells were centrifuged, and pellets resuspended in 50 μl RNase A (1 mg/ml; Sigma-Aldrich) in PBS. After 30 min incubation at room temperature, cells were stained with propidium iodide (PI, p4170; Sigma-Aldrich) and analyzed using a flow cytometer (BD Biosciences). For each measurement, 1 × 10^4^ events were collected, and data were analyzed by Flowlogic software (Inivai Technology).

### Drug interaction analysis

Cells were seeded at 2000 cells/well and allowed to attach for 24 h before drugs or vehicle were added. Cells were treated with increasing doses of palbociclib, dinaciclib, and ET (letrozole or fulvestrant) or an equipotent combination of the inhibitors and incubated at 5% CO_2_ and 37 °C for 96 h. Cell growth was evaluated using crystal violet-based colorimetric assay and interactions were calculated with Compusyn software (ComboSyn, Inc.), based on the combination index (CI) equation from Chou-Talalay method^86^. Drug interaction was scored as follows: CI = 1 is additive, CI < 1 is synergistic, CI > 1 is antagonistic.

### Western blotting

Whole cell extracts were obtained using RIPA buffer (50 mM Tris HCl (pH 8), 150 mM NaCl (pH 8), 1% IgePAL 630, 0.5% sodium deoxycholate, 0.1% SDS) containing protease and phosphatase inhibitors Complete and PhosSTOP (4906837001, Roche). Pierce BCA Protein Assay Kit (23225, Thermo Fisher Scientific) was used to measure the protein concentration of the lysate samples and the OD was measured at 562 nm in the Paradigm microplate reader. Protein lysate (25–30 μg) was loaded on a 4–20% SDS-PAGE gel (Bio-Rad) under reducing conditions and electroblotted onto a PVDF transfer membrane (Bio-Rad). Membranes were blocked in Tris-buffered saline (TBS), 0.1% Tween-20 (Sigma-Aldrich) containing 5% non-fat dry milk powder (Sigma-Aldrich) for 1 h at room temperature. The following primary antibodies were used according to the manufacturer´s protocol: anti-ER (RM-9101-S1, 1:500) and phospho-Rb S612 (PA5-12680, 1:500) from Thermo Fisher Scientific; anti-CDK6 (ab124821, 1:5000), anti-cyclin A2 (ab38, 1:500), anti-p-c-myc S62 (ab51156, 1:1000), and as loading control anti-β-actin (ab6276, 1:500000) from abcam; anti-cyclin E1 (HE12, BDB551159, 1:500) and anti-P27 (610241, 1:5000) from BD Biosciences; anti-CDK2 (2546, 1:1000), anti-p-CDK2 T160 (2561, 1:500), anti-cyclin E2 (4132S, 1:1000), anti-cyclin D1 (2978, 1:1000), anti-cyclin D3 (2936, 1:1000), anti-p-Rb S780 (3590, 1:1000), anti-Rb (9309, 1:1000), anti-p-AKT S473 (4060, 1:1000), anti-AKT (pan) (4685, 1:1000), anti-mTOR (2983, 1:1000), anti-p-mTOR S2448 (2971, 1:1000), anti-p-PRAS40 T246 (2997, 1:1000), anti-PRAS40 (2691, 1:1000), anti-p-S6 S235/236 (2211, 1:1000), anti-S6 (2217, 1:1000), anti-cleaved PARP (9541, 1:500), anti-Bcl-2 (2872, 1:1000), anti-Bax (2772, 1:1000), anti-c-myc (5605, 1:1000), anti-ERK1/2 (9102, 1:1000), anti-p-ERK1/2 Thr202/Tyr204 (4370, 1:2000) and anti-P21 ^waf1/cip1^ (2946, 1:2000) from Cell Signaling; anti-P16 (sc-74401, 1:500), anti-CDK4 (sc-23896, 1:1000), and, as loading control, anti-GAPDH (sc-32233, 1:20000) from Santa Cruz. Secondary antibodies horseradish peroxidase (HRP)-conjugated goat anti-mouse (P0447, Dako, 1:5000) and HRP-conjugated goat anti-rabbit (P0448, Dako, 1:5000) were incubated in blocking buffer for 1 h at room temperature. Membranes were developed with Clarity™ Western ECL Substrate (Bio-Rad) and visualized on Fusion-Fx7-7026 WL/26MX instrument (Vilbaer) with different exposure times, depending on the protein being analyzed. All blots from the same experiment were processed in parallel. Uncropped full blots are shown in Supplementary Figs. 16–18.

### In vivo xenograft studies

Four- to 6-week-old female NOG CIEA mice (Taconic) were ovariectomized and allowed to recover from surgery for 1 week before tumor implantation. Cells for the implantation were cultured in standard medium, washed once with PBS, and 3 × 10^6^ cells resuspended in 50% matrigel (ECM from Engelbreth-Holm-Swarm sarcoma; Sigma-Aldrich), were orthotopically implanted in the mammary fat pads. For primary tumor growth at aromatase-dependent growth conditions, mice were treated daily with subcutaneous injections of sonicated suspension of testosterone (250 μg) and letrozole (10 μg) formulated in 0.5% carboxymethylcellulose (Sigma-Aldrich). The presence of AI letrozole during tumor development is required to maintain AI resistance^87^. Tumors were measured [length (*L*) and width (*W*)], by calipers and the tumor volume was calculated using the formula 0.5 × (*L*) × (*W*)^2^.

When tumors reached a certain size (indicated in the figure legend), tumor-bearing mice were weighed and randomized into four treatment groups (6 mice/group) and treated daily with vehicle (orally with 25% HP-beta-cyclodextrin and intraperitoneally with 5% HP-beta-cyclodextrin), or orally with palbociclib (12.5 mg/kg) dissolved in 25% HP-beta-cyclodextrin alone or in combination with dinaciclib (10 mg/kg) dissolved in 5% HP-beta-cyclodextrin intraperitoneally. Treatments were continued for 5 weeks and tumor volumes were measured weekly. Tumors were harvested at the end of the treatment period for measurement of size and subsequent western blotting analyses.

All animal experiments were approved by The Experimental Animal Committee of The Danish Ministry of Justice and were performed at the animal core facility at University of Southern Denmark. Mice were housed under pathogen-free conditions with ad libitum food and water.

### BC patient samples and clinical endpoints

Formalin-fixed, paraffin-embedded (FFPE), metastatic lesions of ER+ BC patients treated with AI-monotherapy in the advanced setting (*N* = 109) were selected retrospectively by database extraction from the archives of the Department of Pathology at Odense University Hospital (OUH) from 2014 to 2017. Similarly, patients treated with combined CDK4/6i and ET in the advanced setting (*N* = 102) were selected between 2018 and 2019. Tumors were defined as ER+ if ≥ 1% of the tumor cells were stained positive. The inclusion criteria were ER+ BC patients who had undergone surgery or biopsy for advanced-stage disease treated with either AI-monotherapy or combined CDK4/6i and ET in the advanced setting at OUH, and with available clinical information and pathological verification that the metastatic lesion was of BC origin. Exclusion criteria were insufficient tumor material in the FFPE block or incomplete clinical data. These criteria yielded 54 metastatic lesions for the AI-monotherapy-treated cohort and 83 metastatic lesions for the combined CDK4/6i and ET cohort. These metastatic samples were taken before commencing treatment with AI-monotherapy or combined CDK4/6i and ET. All selected samples were coded to maintain patient confidentiality. The study was approved by the Ethics committee of the Region of Southern Denmark (approval no. S-2008–0115) and the Danish Data Protection Agency (approval no. 2008-580035(14/10607)). All tissue samples were collected in compliance with informed consent policy. Progression-free survival (PFS) was defined as the time from the date of starting the respective treatment until disease progression (local or distant relapse) or death.

### Immunocytochemistry and immunohistochemistry

Paraffin-embedded sections (4 μm) from tissue blocks were cut with a microtome. Immunohistochemistry (IHC) was performed using an automated immunostainer (Ventana BenchMark ULTRA, Ventana Medical Systems). Conditions for immunohistochemical analysis for CDK6, phospho-CDK2, and cyclin E1 antibodies were optimized for each antibody regarding method of antigen retrieval and antibody concentration using tissue sections from a tissue microarray (TMA) containing >25 different normal tissues, e.g., tonsil, lung, colon, liver and breast, as well as 10 cancers of different origins. The specificity of the antibodies was also evaluated based on this initial analysis. Paraffin-embedded sections were mounted on ChemMateTM Capillary Gap Slides (Dako), dried at 60 °C, deparaffinized, and hydrated. Prior to antigen retrieval, blocking of endogenous peroxidase was performed in 1.5% hydrogen peroxide in TBS buffer, pH 7.4, for 10 min. A panel of antigen retrieval protocols was initially evaluated, including heat-induced epitope retrieval by microwave boiling for 15 min in (i) in T-EG solution/TRS buffer (Dako), (ii) 10 mM citrate buffer, pH 6.0, or (iii) pretreatment with cell conditioner 1 (CC1) buffer for 32 min at 100 °C or 36 min at 37 °C. Heat-induced epitope retrieval by microwave boiling in T-EG for 15 min proved to be the optimal antigen retrieval method for the CDK6 antibody with additional pretreatment with cell conditioner 1 (CC1) buffer for 32 min at 100 °C for cyclin E1 antibody. For phospho-CDK2, incubation in T-EG solution buffer without heating (MBO) for 15 min was the optimal antigen retrieval method. Sections were subsequently incubated with each of the primary antibodies diluted in antibody diluent (S2022, Dako) for 1 h at room temperature, washed with TNT buffer (0.1 M Tris, 0.15 M NaCl, 0.05% Tween-20, pH 7.5). Primary antibody dilutions used were anti-CDK6 (124821, Abcam) 1:750, anti-p-CDK2 (2561, Cell Signaling Technologies) 1:50, and anti-cyclin E1 (551159, BD Biosciences) 1:100. Primary antibody binding was detected with HRP-conjugated EnVision^TM^ FLEX+ Rabbit (LINKER) (K8009, Dako) with 3,30-diaminobenzidine (DAB) (K3468, Dako) as chromogen for CDK6 antibody, while Optiview-DAB (Ventana Medical Systems) was used for phospho-CDK2 and cyclin E1 antibodies. Immunostaining was followed by nuclear counterstaining in Mayer’s hematoxylin. Finally, coverslips were mounted with AquaTex (Merck). For each experiment, samples with either an isotype-matched antibody or no primary antibody were included as controls, while the TMA was used to evaluate the specificity of each antibody.

Quantification of CDK6, p-CDK2, and cyclin E1 stainings was performed by an experienced breast pathologist in a blinded setup. The histoscore (*H*-score) method was used to quantify CDK6, p-CDK2 and cyclin E1. Briefly, the nuclear staining of these proteins in tumor cells was scored based on the staining intensity (0 to 3) multiplied by the percentage of positive cells, on a scale from 0 to 300. The cutoff values used for high vs. low expression were: CDK6 *H*-score > 0, p-CDK2 H-*s*core ≥ 75, cyclin E1 *H*-score ≥ 100, as determined from the median expression and survival significance of AI-treated patients.

### Statistical analysis

One-way or two-way ANOVA was used for all in vitro and in vivo experiments unless otherwise indicated. Grubbs’ test was used to find and exclude outliers in the in vivo analysis. Survival curves were generated by Kaplan–Meier and log-rank tests to estimate the correlation (hazard ratio “HR” and *p*-value) between CDK6, p-CDK2, and cyclin E1 expression (as single or combined biomarkers) and PFS. Association between the combined biomarker signature and patient clinicopathological parameters was determined by Fisher’s exact and chi-square (*χ*^2^) tests. Uni and multivariate analysis were performed using Cox proportional hazard regression model to assess the adjusted HR of PFS by the combined biomarker signature and clinicopathological characteristics. The *p*-values < 0.05 were considered significant. For statistical analysis, STATA v16.0 (STATACorp) and GraphPad Prism v8 (GraphPad Software, Inc.) were used.

### Reporting summary

Further information on research design is available in the Nature Research Reporting Summary linked to this article.

## Supplementary information

Supplementary data

REPORTING SUMMARY

## Data Availability

The gene expression data generated during the study are publicly available in the gene expression omnibus (GEO) database under the accession number GSE210399. Survival analyses and immunohistochemistry data are not publicly available to protect patient privacy, but will be made available to authorized researchers who have an approved Institutional Review Board application and have obtained approval from. Please contact the corresponding author with data access requests. All other datasets generated during the study will be made available upon reasonable request to the corresponding author, Dr. Henrik Ditzel, email address: hditzel@health.sdu.dk. Uncropped western blots are part of the supplementary information.

## Code availability

No custom code or scripts were created to perform the data analyses in this study. Graphs were made using GraphPad Prism (v8).
