# Supplementary material for: Co-targeting CDK2 and CDK4/6 overcomes resistance to aromatase and CDK4/6 inhibitors in ER+ breast cancer
Source: NPJ Precis Oncol. 2022 Sep 24;6:68. doi: 10.1038/s41698-022-00311-6 (PMC9509389; doi:10.1038/s41698-022-00311-6)
Supplement: Supplementary file 1 — Supplementary data [file 41698_2022_311_MOESM1_ESM.docx]

**Supplementary Data**

**Supplementary Figures**


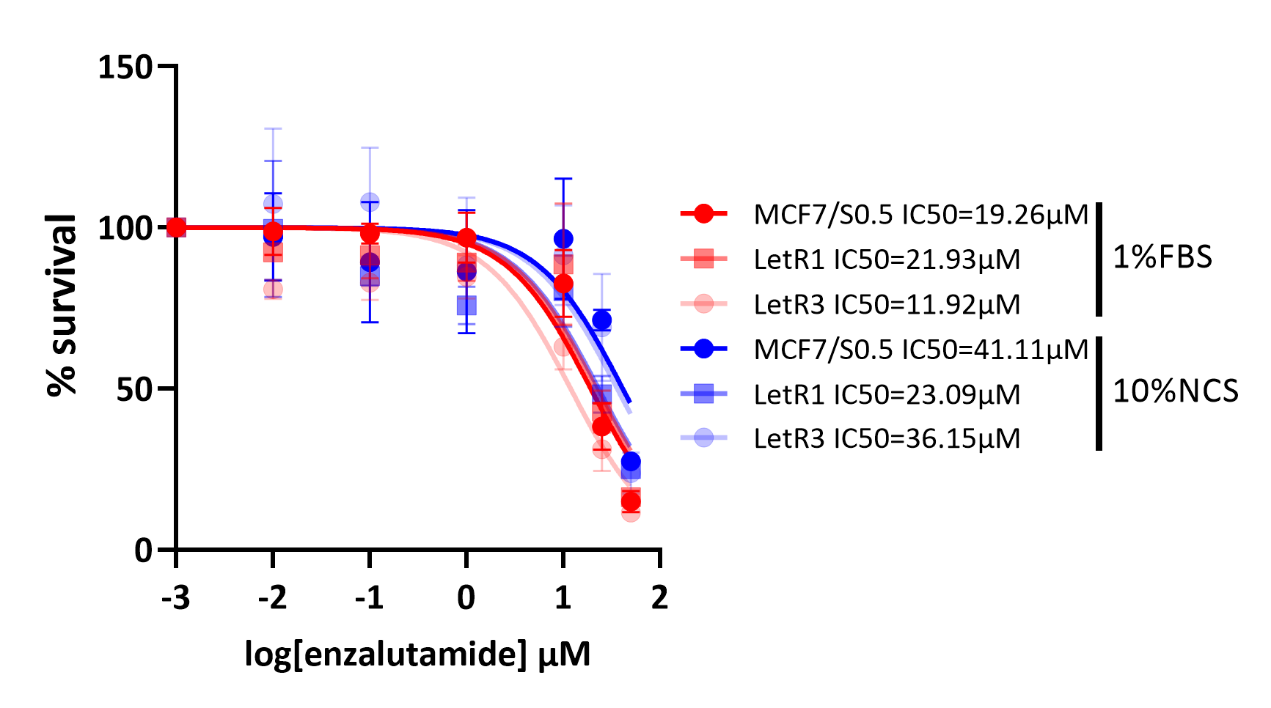


**Supplementary Figure 1. Resistance to letrozole is not associated with androgen** **dependency in LetR cells.** Concentration-dependent cell growth inhibition performed in MCF7/S0.5 and LetR cell lines to determine the IC_50_ of the androgen receptor inhibitor enzalutamide, as assessed by crystal violet assay 6 days after treatment. The data represent the mean of triplicates ± SD.


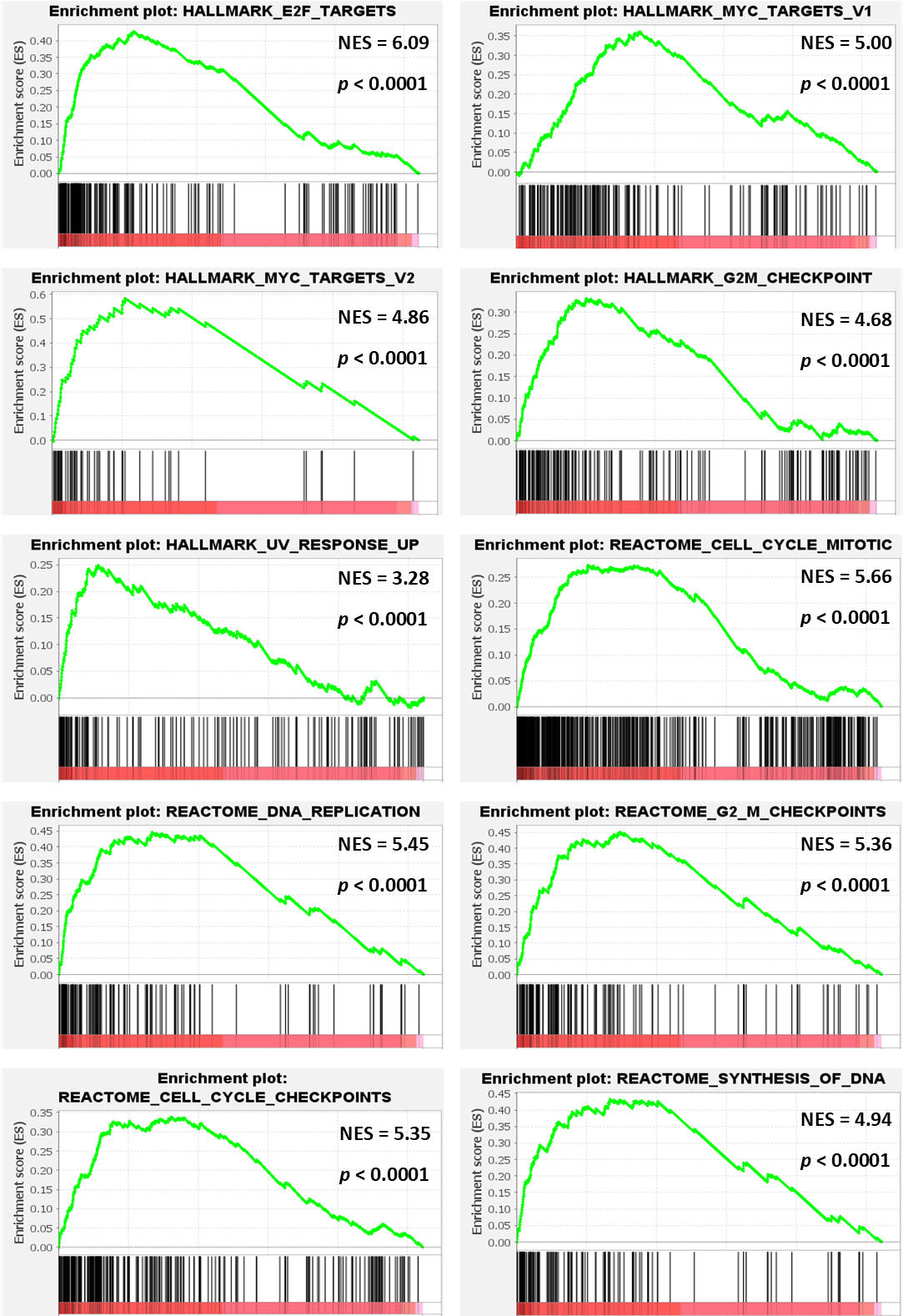


**Supplementary Figure 2.** Gene set enrichment analysis-enrichment plots of representative gene sets from Table S1 enriched in letrozole-resistant cells vs. parental MCF7/S0.5 cell line. FDR, false discovery rate; NES, normalized enrichment score.


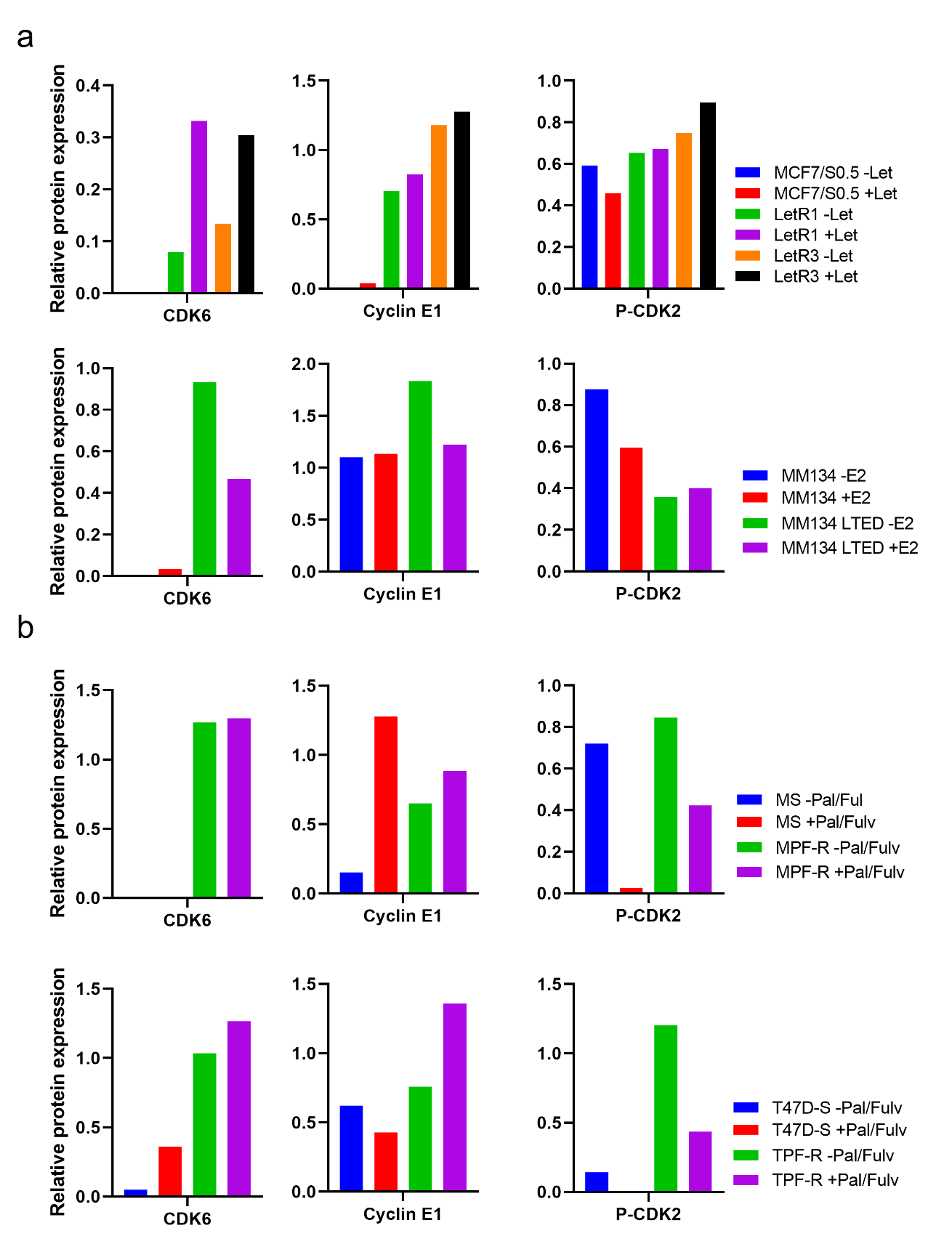


**Supplementary Figure 3. Altered expression of CDK6, Cyclin E1 and p-CDK2 in AI-resistant and combined palbociclib and fulvestrant-resistant ER+ BC cells.** Densitometry analysis of Western blotting bands of CDK6, Cyclin E1 and p-CDK2 proteins in **a** AI-resistant and **b** combined palbociclib and fulvestrant-resistant cell models performed by ImageJ software. Data are normalized to GAPDH (**a**) or β-actin (**b**).


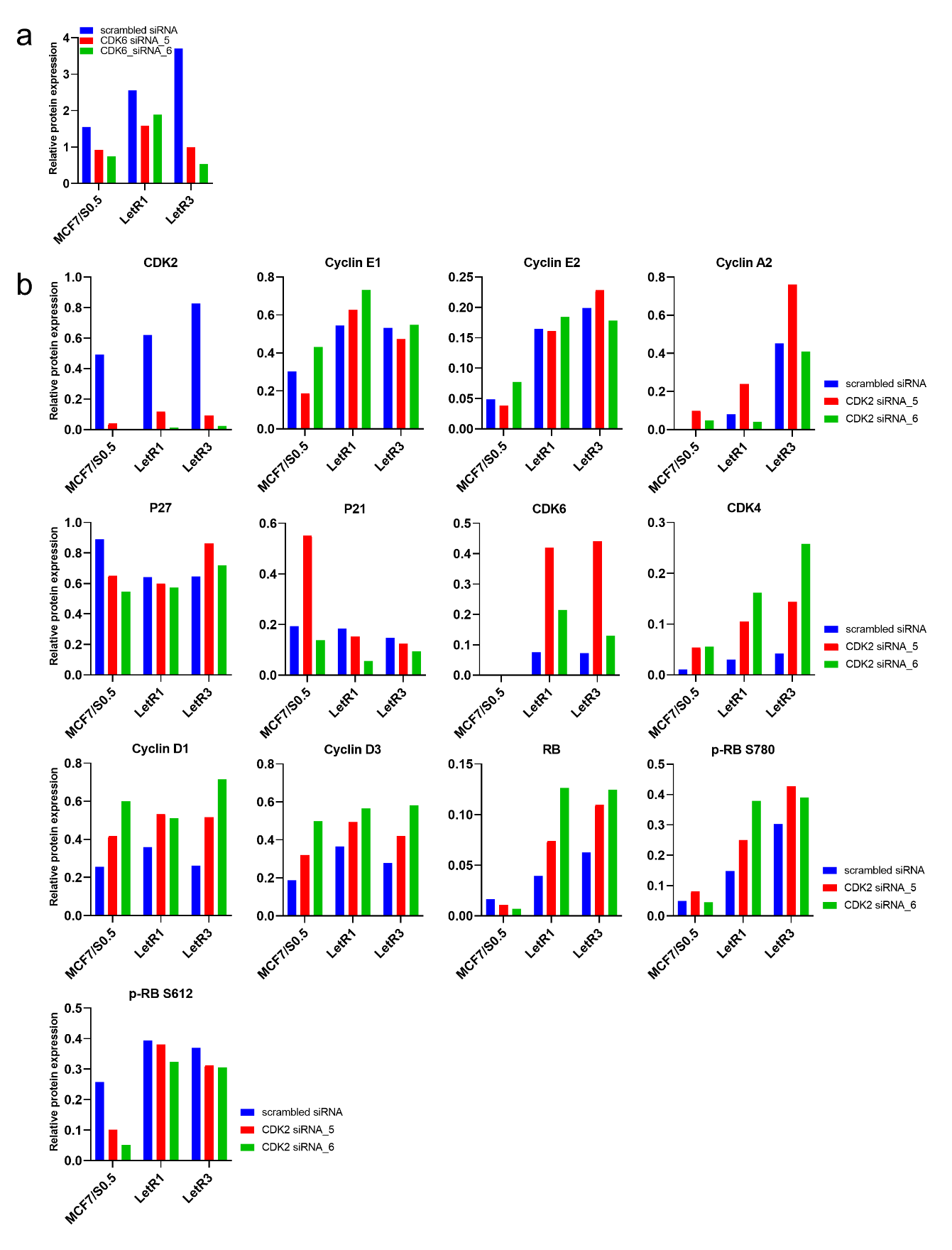


**Supplementary Figure 4. Altered protein expression of the G1/S transition cooperative cascades following CDK2 siRNA-mediated knockdown in letrozole-resistant ER+ BC cells. a** Densitometry analysis of Western blotting bands of CDK6 following CDK6 siRNA-mediated knockdown in letrozole-resistant cell line model. **b** Densitometry analysis of Western blotting bands of the regulators of G1/S transition cooperative cascades following CDK2 siRNA-mediated knockdown in letrozole-resistant cell line model. Densitometry analysis was performed using ImageJ software and normalized to β-actin.


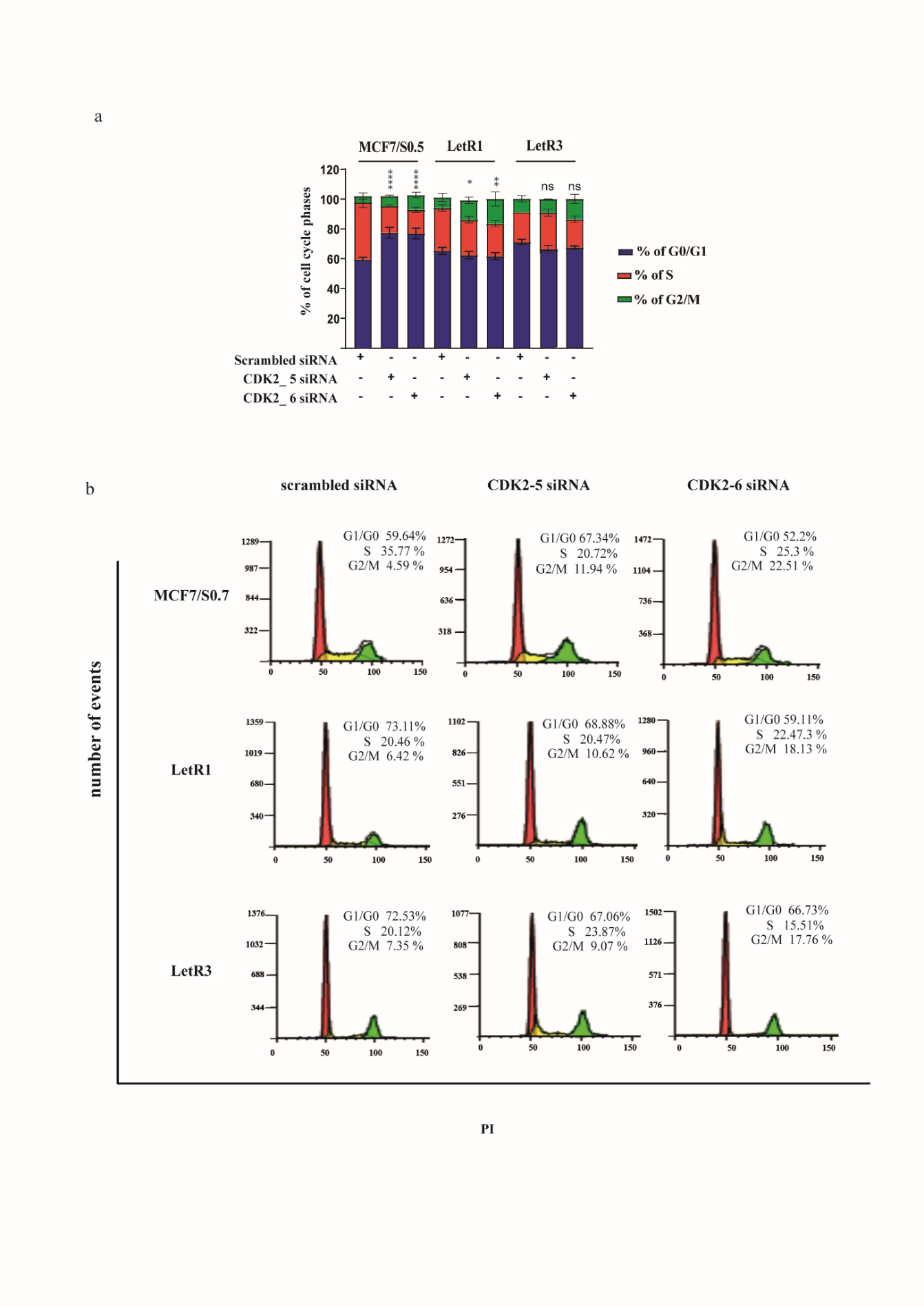


**Supplementary Figure 5. Cell cycle distribution of letrozole-resistant cells after siRNA-mediated CDK2 knockdown.** Letrozole-resistant (LetR) and the parental sensitive cell line MCF7/S0.5 were transfected with 2 CDK2-specific siRNAs (CDK2_5 and CDK2_6), and scrambled siRNA (control). Cell cycle analysis was performed by flow cytometry on propidium iodide-stained cells 96 h after transfection. **a** Cell cycle analysis determining the percentage of cells in the different cell cycle phases. The data represent the mean of triplicates ± SD. The *p* value is calculated using one-way ANOVA test. Atatistical significance differences are shown as ns *p*> 0.05,* *p* ≤ 0.05, and *****p* ≤ 0.0001 for the S-phase. **b** Representative histograms data showing the distribution of transfected cells in the G0/G1, S, and G2/M phases.

**
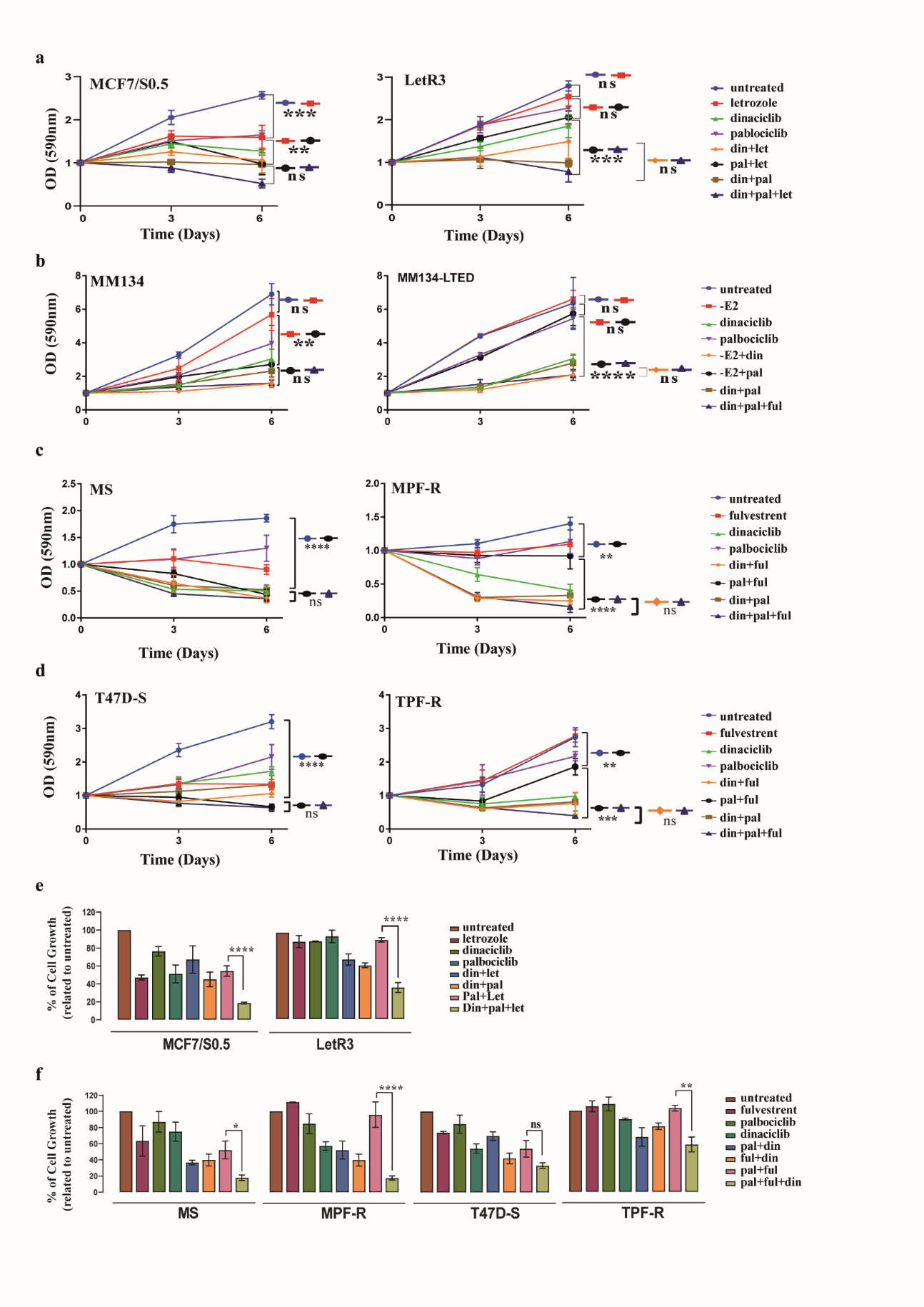
**

**Supplementary Figure 6. CDK2i synergize with CDK4/6i and ET to reduce cell viability in CDK6, p-CDK2, and/or cyclin E1 upregulated resistant ER+ BC cells. a-d** The effect of AI (let, 1μM or absence of E2, 1μg/ml), fulvestrant (ful, 100nM), CDK2i dinaciclib (din, 10nM), or CDK4/6i palbociclib (pal, 150nM) alone or in different combinations was assessed on resistant cells (LetR3, MM134-LTED, MPF-R, and TPF-R) and their parental cell lines (MCF7/S0.5, MM134, MS, T47D-S, respectively), cell viability was assessed by CellTiterBlue and performed over 6 days (data are shown relative to T=0). **e-f** Cell growth assay was performed at 96 h of treatment. Concentrations with sub-inhibitory effect were chosen for the drugs to explore the synergistic potential; 2.5nM din, ET (125nM let or 50nM ful), or 75nM pal alone or in different combination. The data represent the mean of triplicates ± SD. The *p* value is calculated using one-way ANOVA test. Statistically significant differences are shown as ns *p* > 0.05, * *p* ≤ 0.05, ***p* ≤ 0.01, ****p* ≤ 0.001, and *****p* ≤ 0.0001.

**
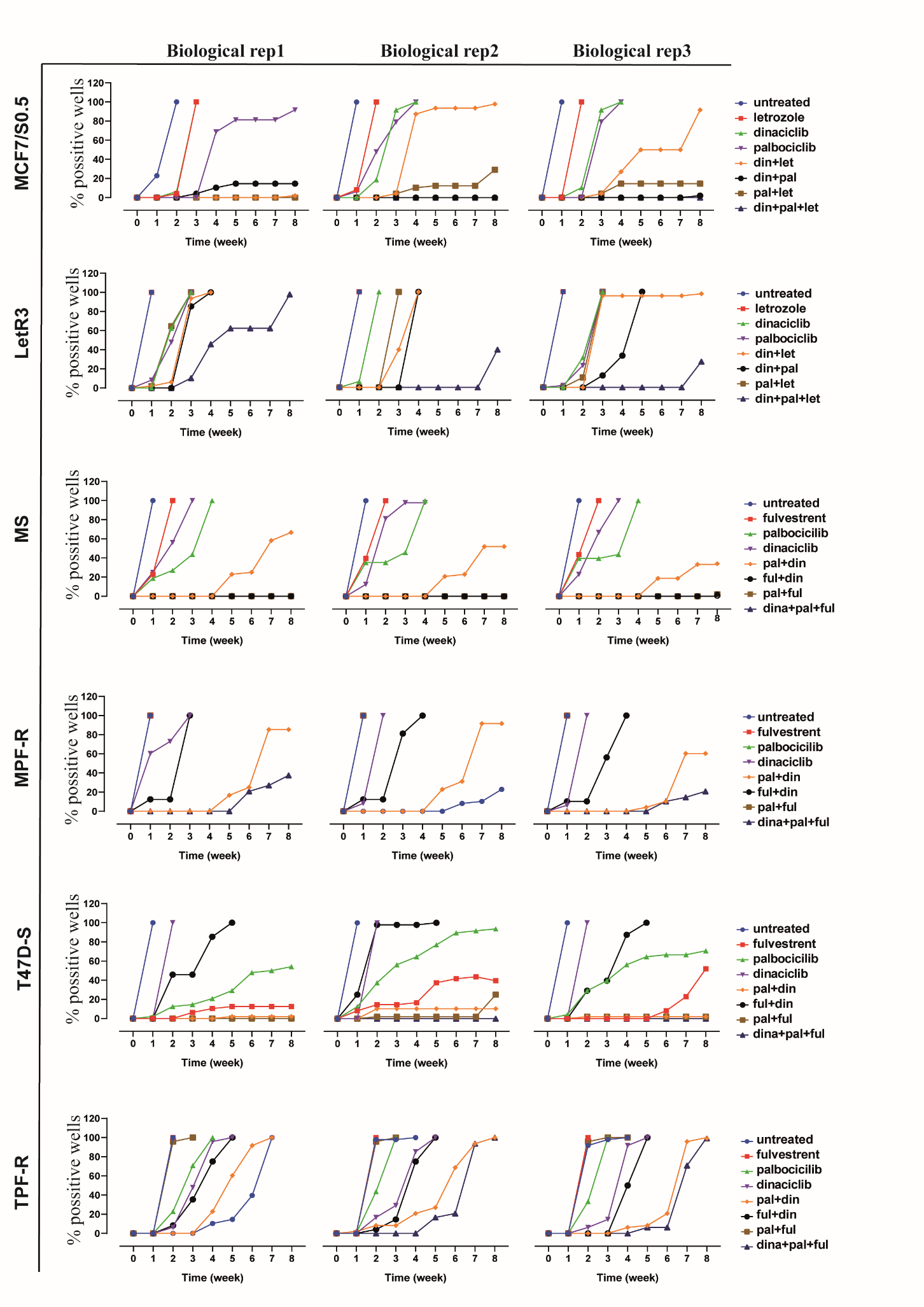
**

**Supplementary Figure 7. Delayed acquisition of resistance by the triple combination of CDK2i, CDK4/6i and ET (letrozole or fulvestrant).** Outgrowth assay data for the three biological replicates/cell line shown as mean in Fig. 3. Resistant cells (LetR3, MPF-R, and TPF-R) and the parental sensitive cell lines (MCF7/S0.5, MS and T47D-S, respectively) were analyzed. Outgrowth of resistant colonies was assessed weekly in 48 wells/treatment condition over 8 weeks. The percentage of positive wells corresponds to the percentage of wells with ≥50% confluency.

**
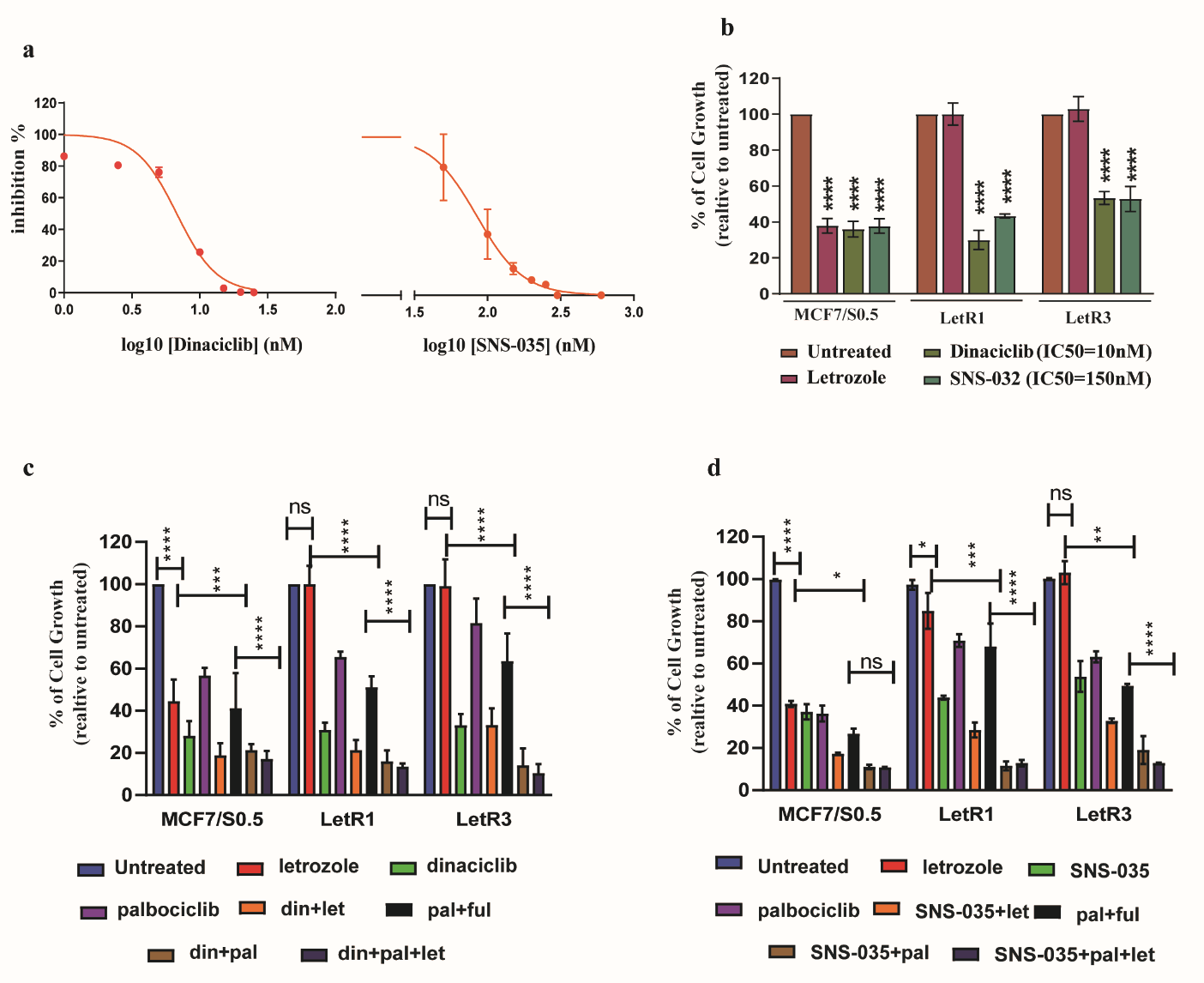
**

**Supplementary Figure 8. The efficiency of the triple combination of CDK2i, CDK4/6i and letrozole is a result of on-target effects. a** Concentration-dependent cell growth inhibition performed on MCF7/S0.5 to determine the IC_50_ of the two CDK2is dinaciclib and SNS-032, as assessed by crystal violet assay 96 h after treatment. **b** Evaluation of the IC_50_ of the two CDK2is dinaciclib and SNS-032 on letrozole-resistant (LetR1, and LetR3) and the sensitive MCF7/S0.5 cells, as assessed by crystal violet assay 96 h after treatment **(c-d),** evaluation of the percentage of cell growth inhibition using the IC_50_ of two CDK2is dinaciclib (din,10nM) and SNS-032 (150nM), letrozole (let, 1μM), and palbociclib (pal, 150nM), as assessed by crystal violet 96 h after treatment. The data represent the mean in triplicates ± SD. The *p* value is calculated using one-way ANOVA analysis. Statistically significant differences are shown as ns *p* > 0.05, **p* ≤ 0.05, ***p* ≤ 0.01, ****p* ≤ 0.001, and *****p* ≤ 0.0001.


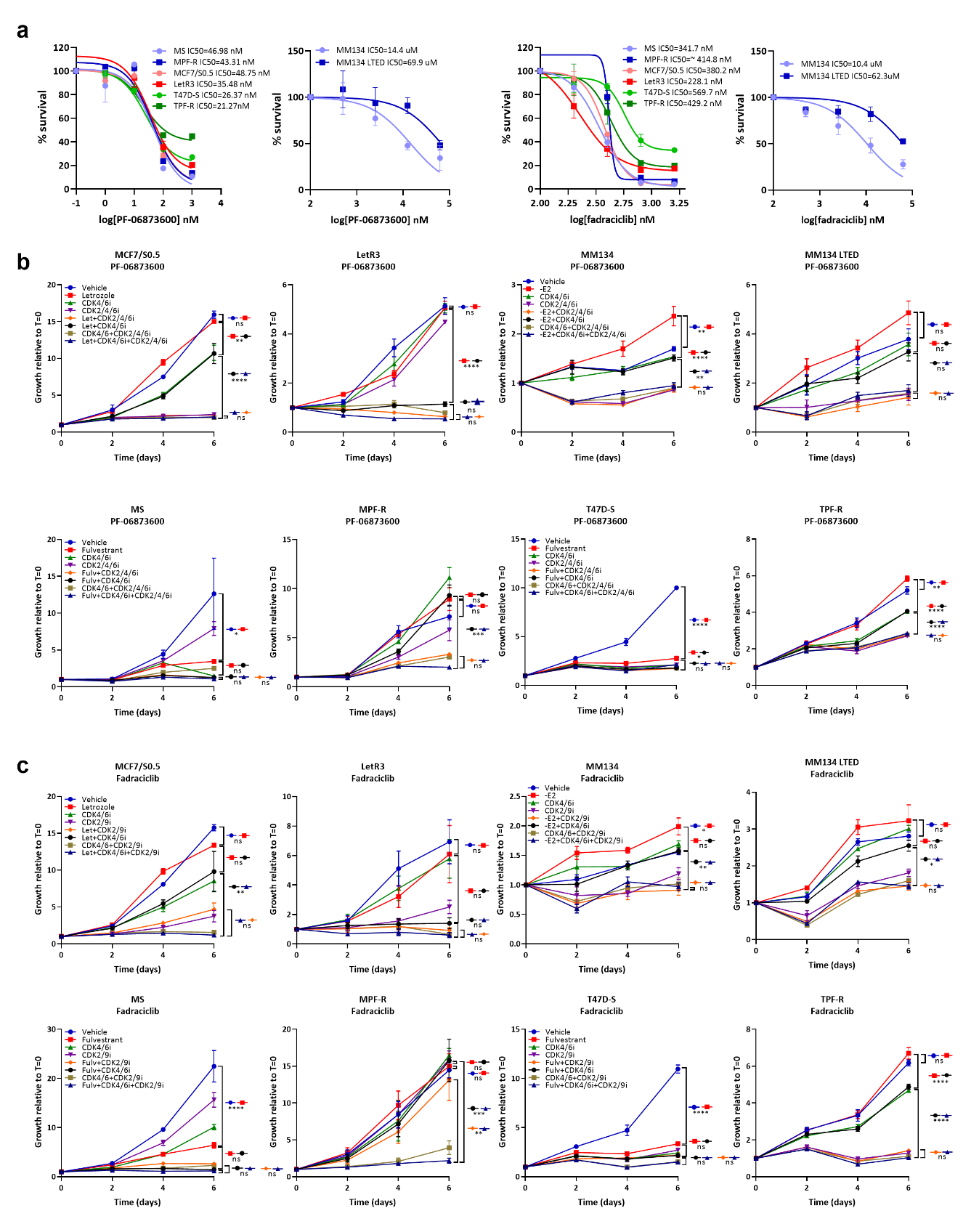
**Supplementary Figure 9. Selective CDK2i fadraciclib (CDK2/9i) and PF-****06873600 (CDK2/4/6i) abrogate growth of AI-resistant and combined CDK4/6i and fulvestrant-resistant ER+ BC cells.** **a** Concentration-dependent cell growth inhibition performed in all cell lines to determine the IC_50_ of the CDK2/4/6i PF-06873600 and the CDK2/9i fadraciclib, as assessed by crystal violet assay 6 days after treatment. Evaluation of the effect of AI letrozole (let, 1μM) or absence of E2 (1μg/ml), fulvestrant (ful, 100nM), CDK4/6i palbociclib (pal, 150nM) and **b** CDK2/4/6i PF-06873600 (50nM in MCF7/S0.5/LetR3 and MS/MPF-R, 100nM in T47D-S/TPF-R, and 50µM in MM134/LTED) or **c** CDK2/9i fadraciclib (300nM in MS/MPF-R, 500nM in MCF7/S0.5/LetR3, 750nM in T47D-S/TPF-R and 50µM in MM134/LTED) alone or in different combinations in resistant cells (LetR3, MM134-LTED, MPF-R, and TPF-R) and parental cell lines (MCF7/S0.5, MM134, MS, and T47D-S, respectively) over 6 days, as assessed by crystal violet.The data represent the mean in triplicates ± SEM. The *p* value is calculated using one-way ANOVA analysis. Statistically significant differences are shown as ns *p* > 0.05, **p* ≤ 0.05, ***p* ≤ 0.01, ****p* ≤ 0.001, and *****p* ≤ 0.0001.


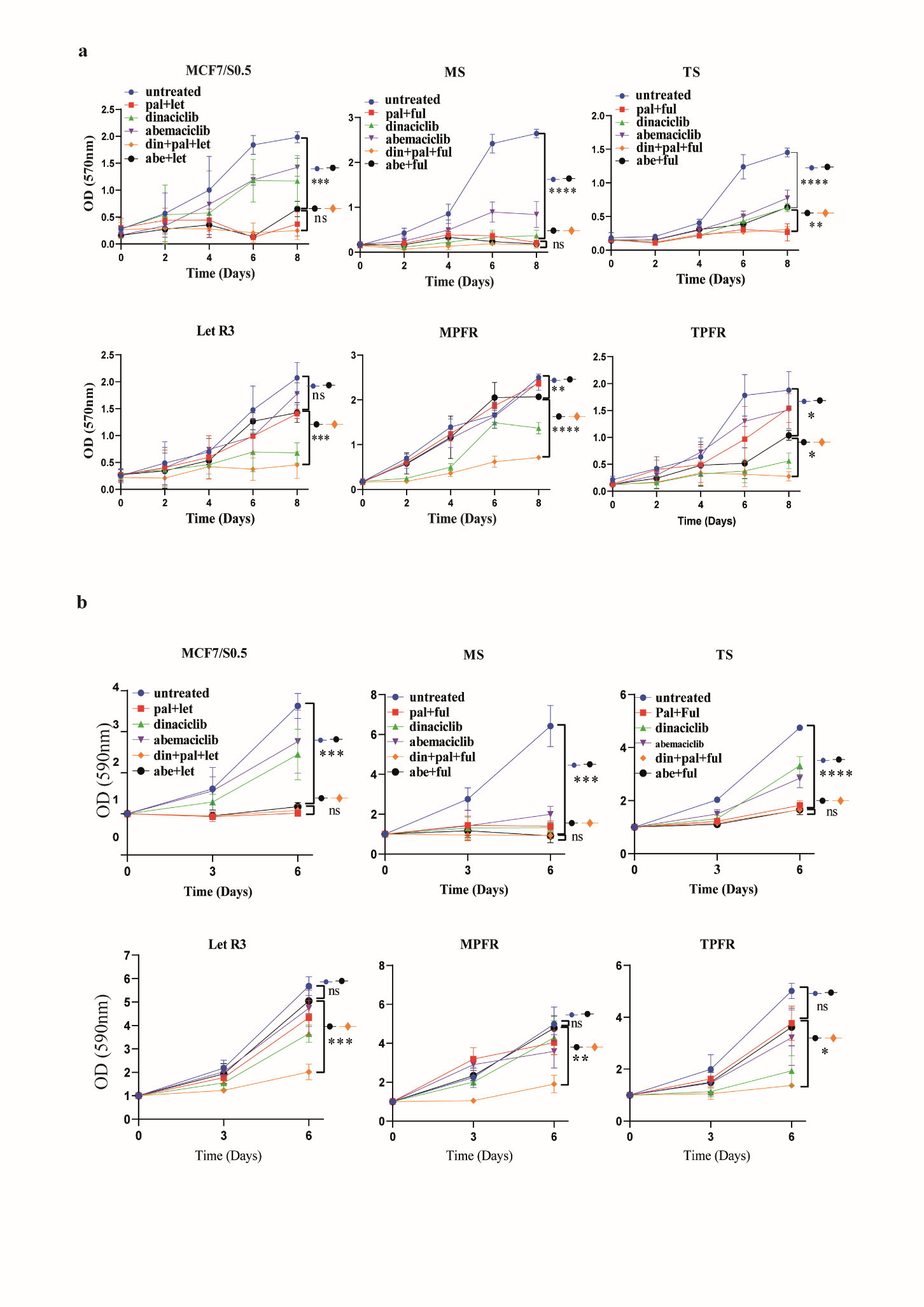


**Supplementary Figure 10. AI-resistant and combined CDK4/6i and ET-resistant cells with high CDK6, p-CDK2 and/or cyclin E1 show cross-resistance to CDK4/6i.** The effect of AI letrozole (let), 1μM or absence of E2, 1μg/ml), fulvestrant (ful, 100nM), CDK2i dinaciclib (din, 10nM ), or CDK4/6i (palbociclib (pal), 150nM or abemaciclib (abe), 150nM] alone or in different combinations evaluated on resistant cells (LetR3, MPF-R, and TPF-R) and the parental cell lines (MCF7/s0.5, MS, and T47D-S, respectively). **a** Evaluation of cell growth over 8 days, as assessed by crystal violet. Data represent the mean of triplicates ± SD. **b** Evaluation of cell viability performed over 6 days, as assessed by CellTiterBlue. Data are relative to T=0 and shown as the mean of triplicates ± SD. The *p* value is calculated using one-way ANOVA test. Statistically significant differences are shown as ns *p* > 0.05, * *p* ≤ 0.05, ***p* ≤ 0.01, and ****p* ≤ 0.001, and *****p* ≤ 0.0001.


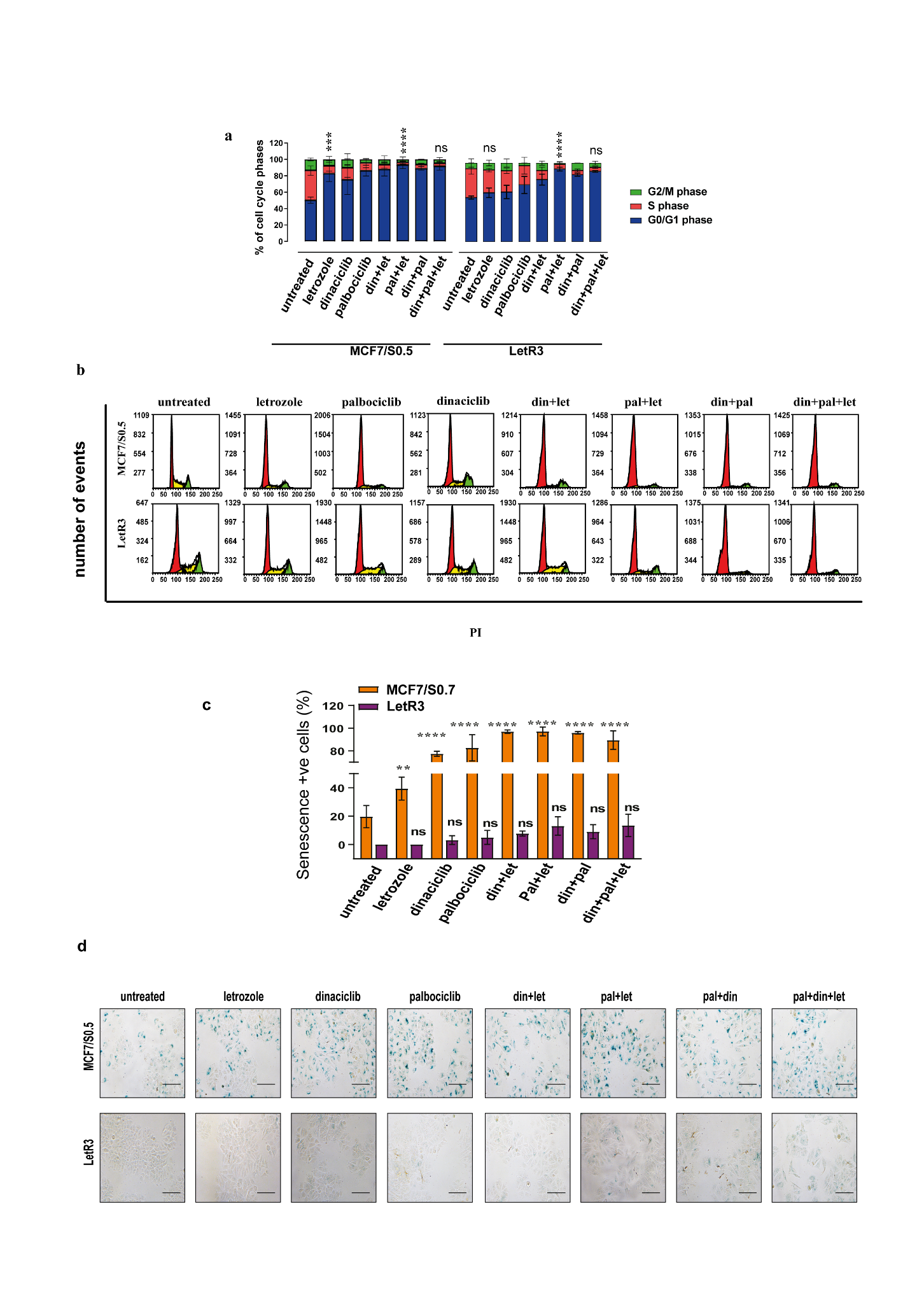


**Supplementary Figure 11. The triple combination of CDK2i, CDK4/6i and AI induced G1/S cell cycle arrest, but not senescence, in letrozole-resistant ER+ BC cells.** The effect of letrozole (1μM), fulvestrant (100nM), CDK2i dinaciclib (10nM), or CDK4/6i palbociclib (150nM) alone or in different combinations was evaluated on letrozole-resistant cells (LetR3) and the sensitive cell lineMCF7S0.5. **a** Cell cycle distribution was examined using flow cytometry on propidium iodide-stained cells 24 h after treatment and is shown as percentage of cells in different cell cycle phases. The data represent the mean of triplicates ± SD. The *p* value is calculated using one-way ANOVA test. Statistically significant differences are shown as ns *p*> 0.05,**p* ≤ 0.05, and ****p* ≤ 0.001 for the S-phase. **b** Representative histograms showing the distribution of treated cells in the G0/G1, S, and G2/M phases. **c** Cell senescence was examined using the senescence-associated SA-ß gal staining kit 6 days after treatment and is shown as percentage of positively-stained cells. Senescent cells were quantified by counting positive cells (200 cells/ filed) in 3 randomly chosen, non-overlapping fields. *P*-values were calculated by one-way ANOVA test. Statistically significant differences are shown as ns *p*> 0.5, and *****p* ≤ 0.0001. Data are shown as the mean of triplicates ±SD. **d** Representative images of senescence-associated SA-ß gel-stained cells 6 days after treatment. Scale bars, 50 µm.


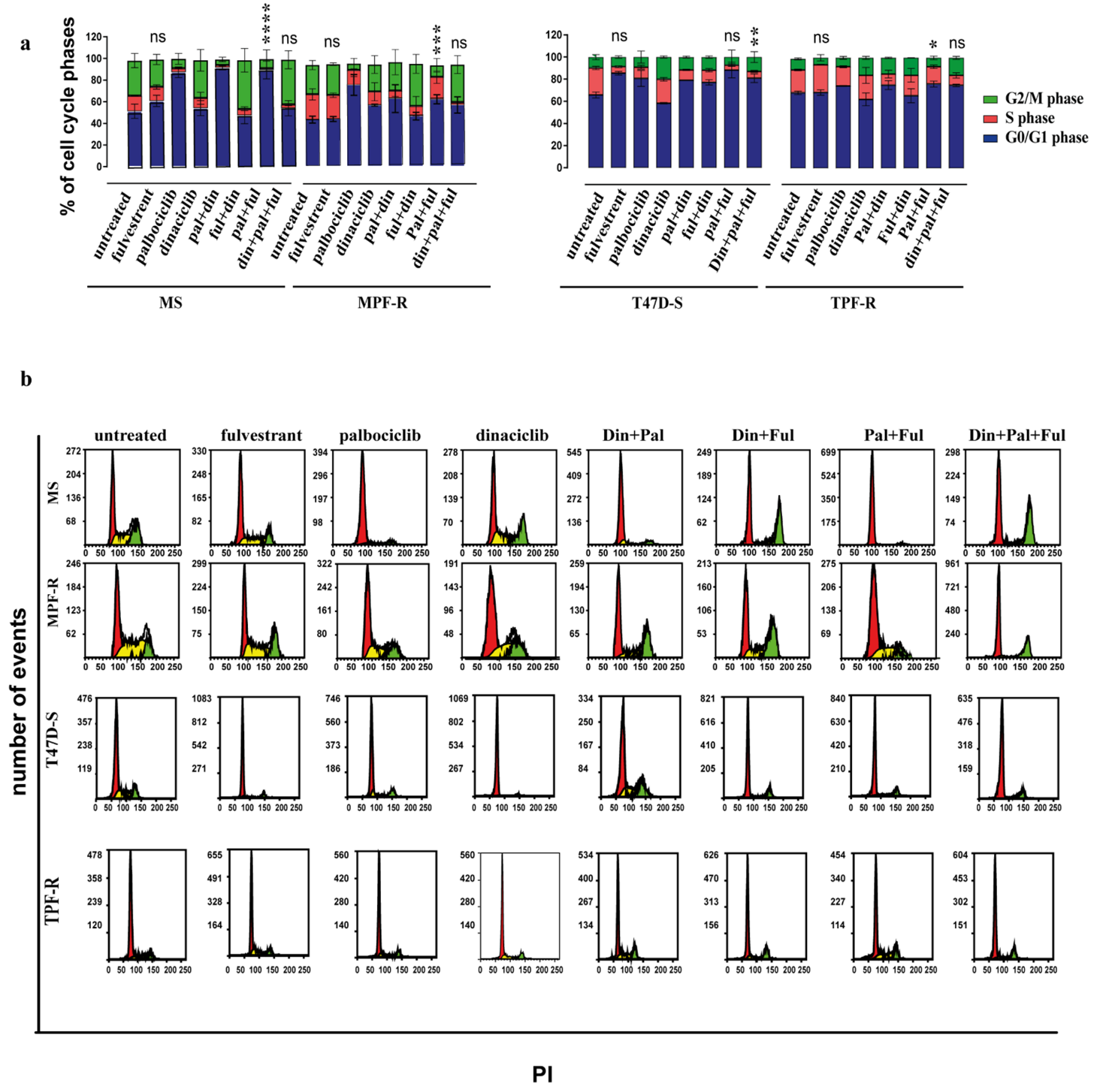


**Supplementary Figure 12. The triple combination of CDK2i, CDK4/6i and fulvestrant induces G2/M cell cycle arrest in combined palbociclib and fulvestrant-resistant ER+ BC cells.** The effect of letrozole (1μM), fulvestrant (100nM), CDK2i dinaciclib (10nM), or CDK4/6i palbociclib (150nM) alone or in different combinations was evaluated on combined palbociclib- and fulvestrant-resistant cells (MPF-R, and TPF-R) and the parental cell lines (MS, and T47D, respectively). **a** Cell cycle distribution was examined using flow cytometry on propidium iodide-stained cells 24 h (MS and MPF-R) and 72 h (T47D-S and TPF-R) after treatment and is shown as percentage of cells in different cell cycle phases. The data represent the mean of triplicates ± SD. The *p* value is calculated using one-way ANOVA test. Statistically significant differences are shown as ns *p*> 0.5, **p* ≤ 0.05, ***p* ≤ 0.01, and ****p* ≤ 0.001, for the S phase. **b** Representative histograms show the distribution of treated cells in the G0/G1, S, and G2/M phases.

**
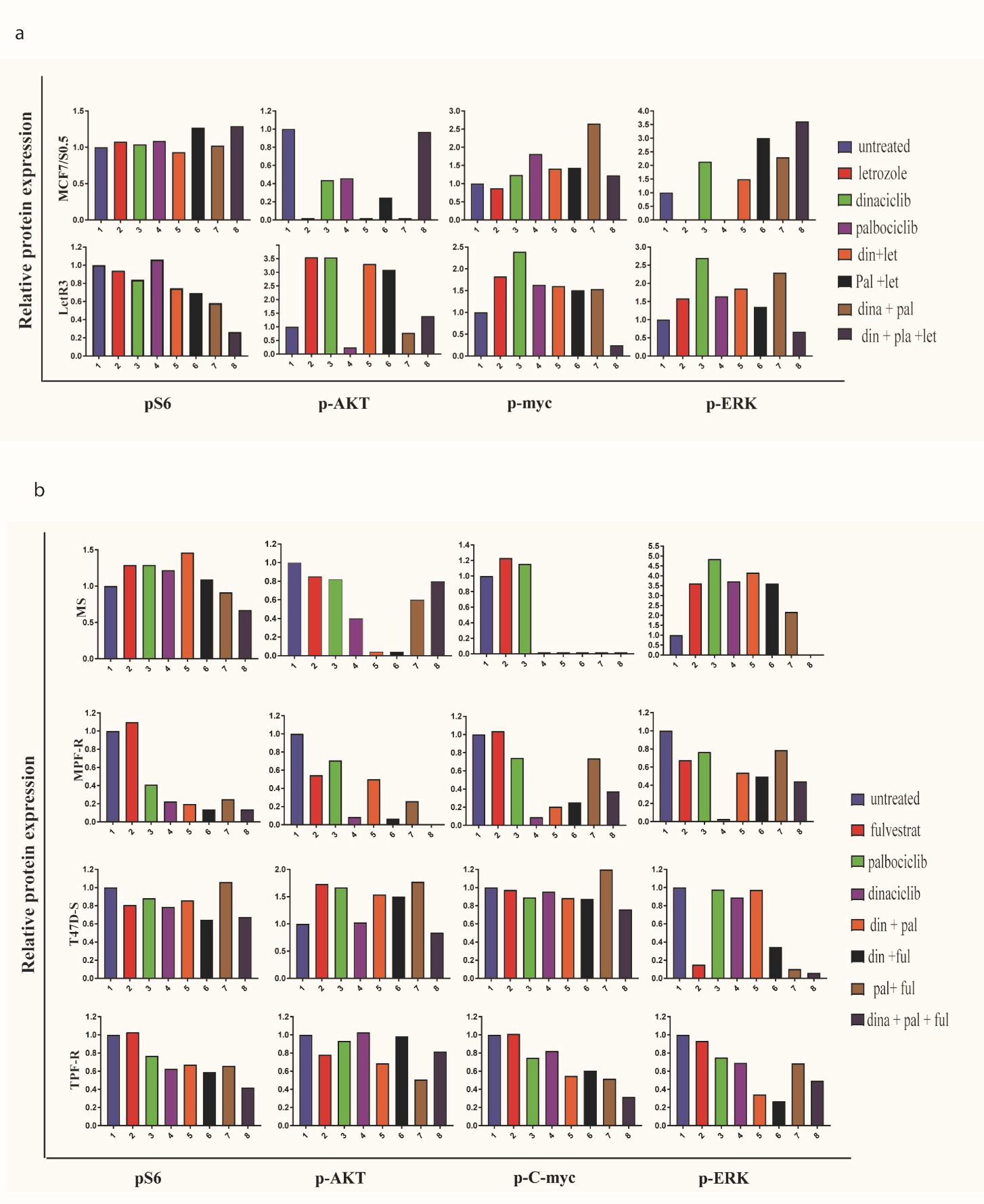
 Supplementary Figure 13. The triple combination of CDK2i, CDK4/6i and ET inhibits the S6 axis by reducing phospho-C-Myc in resistant ER+ BC cells.** Densitometry analysis of Western blotting bands of key transduction signaling proteins was performed by ImageJ software. Data are normalized to β-actin and relative to the control (untreated cells). **a** Letrozole-resistant cells (LetR3) and parental sensitive MCF7S0.5 cells. **b** Combined Palbociclib- and fulvestrant-resistant MPF-R and TPF-R cells and corresponding parental sensitive MS and T47D cells.


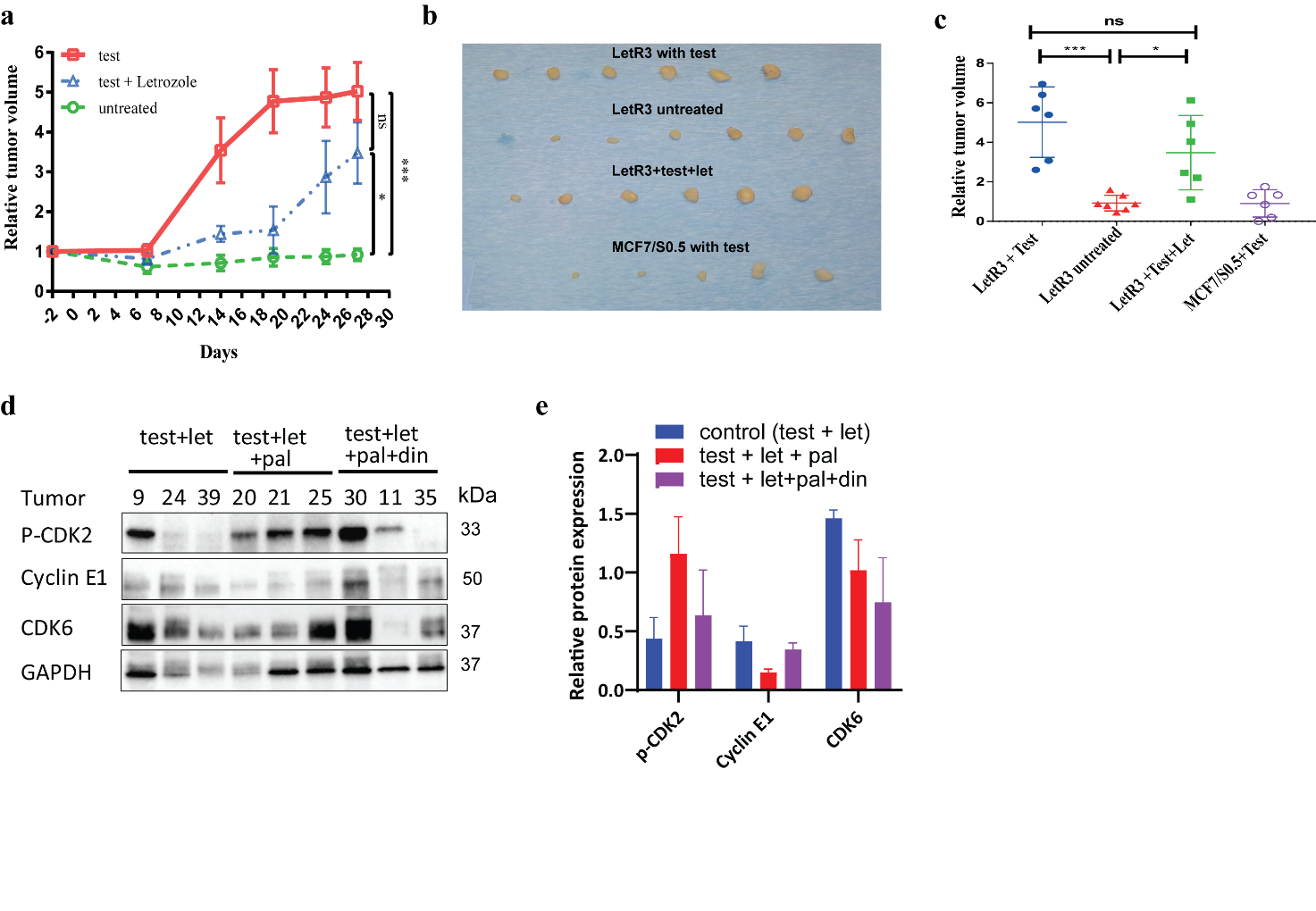
**Supplementary Figure 14. Orthotopic LetR3 tumors grow as an autocrine source of estrogen in a postmenopausal xenograft. a** Tumor growth curves of LetR3-resistant cells under aromatase-dependent conditions [250μg testosterone (test) + 10μg letrozole (let)] relative to treatment initiation (day 0). Data are shown as tumor volume ± SEM. **b** Image of resected tumors from LetR3-resistant cells and MCF7/S0.5 sensitive cells from different treatment groups. **c** Volume of resected LetR3 and MCF7/S0.5 tumors under aromatase-dependent conditions relative to treatment initiation (day 0). Data are shown as mean tumor volume ± SEM. *P* value is calculated using one-way ANOVA test at the endpoint. Statistically significant differences are shown as ns *p*> 0.5, **P* ≤ 0.05 ***P* ≤ 0.01. **d** Western blot analysis of p-CDK2, Cyclin E1 and CDK6 in lysates from 3 tumors of each treatment group. GAPDH was used as loading control. **e** Densitometry analysis of Western blotting bands was performed using ImageJ software and normalized to GAPDH. Data are shown as mean area under the curve (AUC) normalized to the loading control ± SEM.


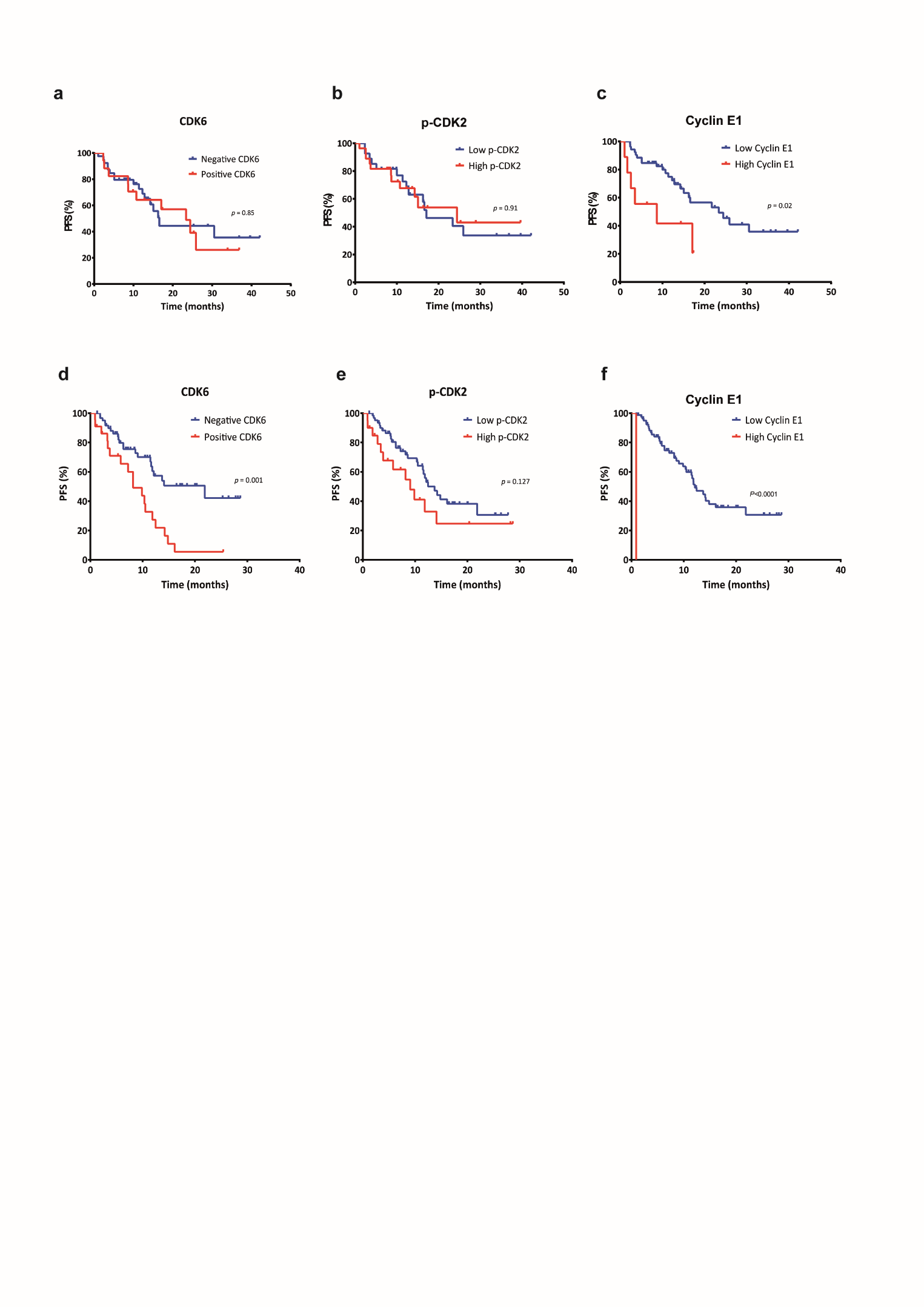


**Supplementary Figure 15. Correlation between CDK6, p-CDK2, and cyclin E1 single proteins levels and PFS in ER+ metastatic BC patients treated with AI-monotherapy or combined CDK4/6i and ET.** Kaplan-Meier plots evaluating progression-free survival (PFS) according to the H-Scoring of CDK6 (cut-off >0), p-CDK2 (cut-off ≥75) and cyclin E1 (cut-off ≥100). **a-c** Cohort of patients receiving AI-monotherapy**. d-f** Cohort of patients treated with **c**ombined CDK4/6i and ET.

**Supplementary Figure 16. Uncropped images of all Western blots shown in Figs. 1 and 2.**

**Supplementary Figure 17. Uncropped images of all Western blots shown in Fig. 4.**

**Supplementary Figure 18. Uncropped images of all Western blots shown in Figs. 5 and 6.**

**Supplementary Tables**

**Supplementary Table 1.** Gene set enrichment analysis in letrozole-resistant LetR cell lines vs. -sensitive parental cell line MCF7/S0.5

|  | Gene set Hallmarks | Size | ES | NES | P value | FDR | Genes |
| --- | --- | --- | --- | --- | --- | --- | --- |
| 1 | HALLMARK_E2F_TARGETS | 189 | 0.43 | 6.09 | 0.000 | 0.000 | *CCNE1, CDK4, CDK1, CDC25A, DONSON, TFRC, TK1, GINS1, RPA1, TRIP13* |
| 2 | HALLMARK_MYC_TARGETS_V1 | 185 | 0.36 | 5.00 | 0.000 | 0.000 | *CDK2, CDK4, CCNA2, MCM5, PCNA, CTPS1, MCM4, RFC4, MCM2, TFDP1* |
| 3 | HALLMARK_MYC_TARGETS_V2 | 55 | 0.58 | 4.86 | 0.000 | 0.000 | *CDK4, HK2, PPRC1, GRWD1, MCM5, UTP20, BYSL, MCM4, UNG, TMEM97* |
| 4 | HALLMARK_G2M_CHECKPOINT | 185 | 0.33 | 4.68 | 0.000 | 0.000 | *CDK1, CDK4, MARCKS, CDC25A, CDC6, MCM5, CCNA2, E2F1, E2F2, E2F4* |
| 5 | HALLMARK_UV_RESPONSE_UP | 155 | 0.33 | 4.68 | 0.000 | 0.000 | *CCNE1, CDK2, CCND3, LYN, FEF18, RET, TAP1, TFRC, CHRNA5, TYRO3* |
|  | **Gene set Reactome** | **Size** | **ES** | **NES** | **P value** | **FDR** |  |
| 1 | REACTOME_CELL_CYCLE_MITOTIC | 478 | 0.27 | 5.66 | 0.000 | 0.000 | *CCNE1, CCNE2, CDK6, CDK2, CDK1, CDK4, LYN, CCND3, CDC25A, ORC1* |
| 2 | REACTOME_DNA_REPLICATION | 127 | 0.45 | 5.45 | 0.000 | 0.000 | *CCNE1, CCNE2, CDK2, CCNA2, ORC1, CDC6, GMNN, GINS1, RPA1, MCM5* |
| 3 | REACTOME_G2_M_CHECKPOINTS | 125 | 0.45 | 5.36 | 0.000 | 0.000 | *CDK2, CDK1, CCNB2, CDC25A, ORC1, CDC6, RPA1, MCM5, MCM10, MCM8* |
| 4 | REACTOME_CELL_CYCLE_CHECKPOINTS | 241 | 0.34 | 5.35 | 0.000 | 0.000 | *CCNE1, CCNE2, CDK2, CDK1, CLSPN, CDC25A, CCNB2, CCNA2, ORC1, CDC6* |
| 5 | REACTOME_SYNTHESIS_OF_DNA | 116 | 0.43 | 4.94 | 0.000 | 0.000 | *CCNE1, CCNE2, CDK2, CCNA2, ORC1, CDC6, GINS1, RPA1, MCM5, GINS2* |

**Supplementary Table 2.** Ingenuity-based biologic interpretation of genes differentially expressed in letrozole-resistant LetR cell lines vs. -sensitive parental cell line MCF7/S0.5: canonical pathways and network functions

| Canonical pathway | P value | | Genes |
| --- | --- | --- | --- |
| Cell Cycle Control of Chromosomal Replication | 4.36E-07 | | *CCNA2, CCNE1, CCNE2, CDK1, CDK2, E2F7, E2F8, ESR1, RBL1, TFDP* |
| Estrogen-mediated S-phase Entry | 1.98E-06 | | *CDC6, CDK1, CDK2, CDK6, DBF4, DNA2, MCM4, MCM5, MCM6, MCM8, ORC1, ORC6, PCNA, POLA1, RPA1* |
| Aryl Hydrocarbon Receptor Signaling | 2.28E-06 | | *AHR,ALDH3B2,ALDH7A1,CCNA2,CCNE1,CCNE2,CDK2,CDK6, ESR1, FOS, GSTA4, GSTM3,GSTM4,GSTT1,MDM2,MGST2,NFKB1,NQO1,POLA1,RARGRBL1,TFDP1,TGFB1,TGFB2* |
|  | | | |
| Molecules in network | **Score** | **Focus molecules** | **Top function** |
| Cell Death and Survival, Cell Cycle, Cellular Growth and Proliferation | 39 | 35 | *AR,BAG3,BCL2,BDKRB2,CAPN1,CASP8,CCNE1,CCNE2,CDC6,CDC25A,CDK1,CDK2,CDK6,DICER1,DUSP10,E2F1,E2F4,GADD45A,MALT1,MAOA,MAPT,MCM4,MYB,MYBL2,NFKB1,NFKBIA,PCNA,PEA15,PGR,POLA1,PRKCD,RBL1,RRM2,SMAD7* |
| Cell Cycle, DNA Replication, Recombination, and Repair, Organismal Survival | 39 | 35 | *ACACA,BHLHE40,BMP1,BMP7,CCNA2,CCNE1,CCNE2,CDC25C,CDK1,CDK2,CDK6,CHUK,CITED2,COL3A1,FASN,FEN1,FURIN,HEXB,MCM4,MYBL2,NRP1,PCNA,RAB31,RAD18,RAD51AP1,RAD51C,RBL1,RFC4,RHOD,SCD,SMC2,SOX4,SREBF1,TGFB1,TIMP1* |

**Supplementary Table 3.** Gene expression alterations of the G1/S cell cycle transition in letrozole-

resistant (LetR) vs. -sensitive (MCF-7/S0.5) cells, as determined by gene array

| G1/S Transition Genes  (encoded protein) | LetR1 vs MCF7/S0.5 | | LetR3 vs MCF7/S0.5 | |
| --- | --- | --- | --- | --- |
|  | **Fold Change** | **P-value** | **Fold Change** | **P-value** |
| *CDK2* | 2.68 | 3.2x10^-7^ | 3.22 | 1.3x10^-7^ |
| *CDK6* | 2.02 | 0.0109 | 2.09 | 0.0011 |
| *CDK4* | 1.2 | 0.0348 | 1.33 | 0.0284 |
| *CCNE1* (Cyclin E1) | 4.71 | 1.5x10^-10^ | 5.07 | 1.2x10^-10^ |

**Supplementary Table 4:** Automated computer simulation of the drug combination index (CI) calculated for dinaciclib, palbociclib and ET (letrozole or fulvestrant) double and triple combinations using CompuSyn program. The doses and the multi-drug effect at 50% cell inhibition after 72 h drug exposure, assessed by crystal-violet colorimetric assay. The effect was classified as synergistic (CI<1), additive (CI=1), or antagonistic (CI>1)

| Cell lines | Drug Combination and doses (nM) | | | CI | Interaction | |
| --- | --- | --- | --- | --- | --- | --- |
|  | **Dinaciclib** | **Palbociclib** | **Letrozole** |  | |  |
| MCF7/S0.5 | 0.02347 | 0.35212 | 1.17374 | **0.02731** | | **Synergistic** |
|  | 4.30777 | 64.6166 | - | **1.26691** | | **Additive** |
|  | 6.86034 | - | 343.017 | **1.538101** | | **Additive** |
|  | - | 5.93871 | 19.7957 | **0.36546** | | **Synergistic** |
|  | | | | | | |
| LetR1 | 1.00237 | 15.0356 | 50.1187 | **0.11169** | | **Synergistic** |
|  | 1.24954 | 18.7430 | - | **0.13914** | | **Synergistic** |
|  | 2.36636 | - | 118.318 | **0.24766** | | **Synergistic** |
|  | - | 9242.35 | 30807.8 | **4.21826** | | **Antagonistic** |
|  | | | | | | |
| LetR3 | 1.76237 | 26.4356 | 88.1186 | **0.18174** | | **Synergistic** |
|  | 3.15062 | 47.2593 | - | **0.32471** | | **Synergistic** |
|  | 2.93543 | - | 146.772 | **0.23985** | | **Synergistic** |
|  | - | 2380.38 | 7934.59 | **3.40671** | | **Antagonistic** |
| Cell lines | Drug Combination and doses (nM) | | | **CI** | | **Interaction** |
|  | **Dinaciclib** | **Palbociclib** | **Fulvestrant** |  |  |  |
| MS | 0.0415 | 0.622 | 0.414 | **0.007** | | **Synergistic** |
|  | 8.783 | 131.7 | - | **0.0616** | | **Synergistic** |
|  | 1.492 | - | 14.916 | **0.253** | | **Synergistic** |
|  | - | 29.11 | 19.40 | **0.332** | | **Synergistic** |
|  | | | | | | |
| MPF-R | 2.854 | 42.818 | 28.54 | **0.0129** | | **Synergistic** |
|  | 8.325 | 124.88 |  | **0.0354** | | **Synergistic** |
|  | 119.2 |  | 1192.5 | **0.495** | | **Synergistic** |
|  |  | 58178 | 87267.1 | **3.71** | | **Antagonistic** |
|  | | | | | | |
| T47D-S | 1.81 | 27.13 | 18.087 | **0.439** | | **Synergistic** |
|  | 8.65 | 129.8 | - | **0.541** | | **Synergistic** |
|  | 5.079 | - | 50.79 | **1.11** | | **Additive** |
|  | - | 42.9 | 28.61 | **0.55** | | **Synergistic** |
|  | | | | | | |
| TPF-R | 4.91 | 73.759 | 49.172 | **0.0387** | | **Synergistic** |
|  | 9.9 | 148.56 | - | **1.18** | | **Synergistic** |
|  | 8.15 | - | 81.54 | **0.978** | | **Additive** |
|  | - | 3415864 | 2277243 | **1.3E-14** | | **Synergistic** |

**Supplementary Table 5.** Clinical and pathological characteristics of the patient cohorts having advanced ER+ breast cancer and treated with AI-monotherapy or combined CDK4/6i and ET according to the level of CDK6, P-CDK2, and/or cyclin E1

| **Parameters** | **Patients treated with AI** | | | | |  | **Patients treated with CDK4/6i + ET** | | | | | |
| --- | --- | --- | --- | --- | --- | --- | --- | --- | --- | --- | --- | --- |
|  | **CDK6, p-CDK2 and /or cyclin E1** | | | | | | | | | | | |
|  | **Low signature** | **High signature** | | **N** | **P value^a^** |  | **Low signature** | **High signature** | | | **N** | **P value^a^** |
| **Age at primary tumor** | | | | | | | | | | | | |
| ≤50 years | 11 | 3 | | 14 | >0.999 |  | 27 | 3 | | | 30 | 0.663 |
| >50 years | 30 | 10 | | 40 |  |  | 50 | 3 | | | 53 |  |
| **Size (mm) primary tumor** | | | | | | | | | | | | |
| ≤20 | 16 | 5 | | 21 | 0.954 |  | 41 | 2 | | | 43 | 0.107 |
| 20-50 | 11 | 4 | | 15 |  |  | 27 | 2 | | | 29 |  |
| >50 | 2 | 1 | | 3 |  |  | 8 | 1 | | | 9 |  |
| Unknown | 12 | 3 | | 15 |  |  | 1 | 1 | | | 2 |  |
| **Lymph node status primary tumor** | | | | | | | | | | | | |
| Positive | 9 | 3 | | 12 | 0.788 |  | 50 | 4 | | | 54 | >0.999 |
| Negative | 22 | 8 | | 30 |  |  | 20 | 1 | | | 21 |  |
| Unknown | 10 | 2 | | 12 |  |  | 7 | 1 | | | 8 |  |
| **Grade primary tumor** | | | | | | | | | | | | |
| I | 5 | 0 | | 5 | 0.179 |  | 23 | 1 | | | 24 | 0.295 |
| II | 10 | 7 | | 17 |  |  | 28 | 1 | | | 29 |  |
| III | 3 | 1 | | 4 |  |  | 19 | 3 | | | 22 |  |
| Unknown | 23 | 5 | | 28 |  |  | 7 | 1 | | | 8 |  |
| **Total** | **41** | | **13** | **54** | |  | **77** | | **6** | **83** | | |

*a* χ2 or Fisher’s exact test

**Supplementary Table 6.** Univariate and multivariate analysis of PFS according to the level of CDK6, p-CDK2, and/or cyclinE1 and clinicopathological characteristics of the metastatic disease of ER+ breast cancer patients treated with AI-monotherapy or combined CDK4/6i and ET

| **Variable** | **AI treated patients** | | | | |  | **CDK4/6i + ET treated patients** | | | | | |
| --- | --- | --- | --- | --- | --- | --- | --- | --- | --- | --- | --- | --- |
|  | **Univariate** | |  | **Multivariate** | |  | **Univariate** | |  | **Multivariate** | | |
|  | **HR (95%CI)** | ***P* value** |  | **HR (95%CI)** | ***P* value** |  | **HR (95%CI)** | ***P* value** |  | **HR (95%CI)** | ***P* value** |  |
| **CDK6, p-CDK2, and/or cyclin E1** | 2.81 (1.25-6.35) | 0.013* |  | 2.99 (1.26-7.12) | 0.013* |  | 4.84 (1.82-12.88) | 0.002* |  | 5.31 (1.70-16.60) | 0.004* |  |
| **Endocrine status** | 1.01 (0.58-1.76) | 0.970 |  | 0.93 (0.37-2.33) | 0.883 |  | 1.84 (1.16-2.90) | 0.009* |  | 1.65 (0.90-3.05) | 0.108 |  |
| **Line of therapy** | 1.01 (0.30-3.39) | 0.991 |  | 1.34 (0.21-8.43) | 0.757 |  | 2.5 (1.32-4.75) | 0.005* |  | 1.61 (0.73-3.56) | 0.239 |  |
| **Age at starting the treatment** | 0.98 (0.23-4.20) | 0.980 |  | 0.97 (0.16-6.02) | 0.975 |  | 0.5 (0.22-1.14) | 0.098 |  | 1.10 (0.42-2.86) | 0.848 |  |
| **Site of relapse** | 1.07 (0.623-1.84) | 0.802 |  | 0.93 (0.47-1.85) | 0.837 |  | 1.04 (0.65-1.67) | 0.871 |  | 1.12 (0.71-1.77) | 0.612 |  |

**P* value ≤ 0.05 considered significant
