# Supplementary material for: Co-targeting CDK2 and CDK4/6 overcomes resistance to aromatase and CDK4/6 inhibitors in ER+ breast cancer
Source: NPJ Precis Oncol. 2022 Sep 24;6:68. doi: 10.1038/s41698-022-00311-6 (PMC9509389; doi:10.1038/s41698-022-00311-6)
Supplement: Supplementary file 2 — REPORTING SUMMARY [file 41698_2022_311_MOESM2_ESM.pdf]

nature portfolio

Corresponding author(s): *Henrik Ditzel and Carla Alves*

Last updated by author(s): *May 10, 2022*

Reporting Summary

Nature Portfolio wishes to improve the reproducibility of the work that we publish. This form provides structure for consistency and transparency in reporting. For further information on Nature Portfolio policies, see our Editorial Policies and the Editorial Policy Checklist.

Statistics

For all statistical analyses, confirm that the following items are present in the figure legend, table legend, main text, or Methods section.

n/a	Confirmed	
☐	☒	The exact sample size (n) for each experimental group/condition, given as a discrete number and unit of measurement
☐	☒	A statement on whether measurements were taken from distinct samples or whether the same sample was measured repeatedly
☐	☒	The statistical test(s) used AND whether they are one- or two-sided<br>*Only common tests should be described solely by name; describe more complex techniques in the Methods section.*
☐	☒	A description of all covariates tested
☐	☒	A description of any assumptions or corrections, such as tests of normality and adjustment for multiple comparisons
☐	☒	A full description of the statistical parameters including central tendency (e.g. means) or other basic estimates (e.g. regression coefficient) AND variation (e.g. standard deviation) or associated estimates of uncertainty (e.g. confidence intervals)
☐	☒	For null hypothesis testing, the test statistic (e.g. F, t, r) with confidence intervals, effect sizes, degrees of freedom and P value noted<br>*Give P values as exact values whenever suitable.*
☒	☐	For Bayesian analysis, information on the choice of priors and Markov chain Monte Carlo settings
☐	☒	For hierarchical and complex designs, identification of the appropriate level for tests and full reporting of outcomes
☒	☐	Estimates of effect sizes (e.g. Cohen's d, Pearson's r), indicating how they were calculated

Our web collection on statistics for biologists contains articles on many of the points above.

Software and code

Policy information about availability of computer code

Data collection	Flowlogic software (Inivai Technology)
Data analysis	STATA v16.0 (STATACorp), GraphPad Pism v8 (GraphPad Software, Inc.), ImageJ Java v8 (NIH), Flowlogic software (Inivai Technology), Flowlogic software (Inivai Technology).

For manuscripts utilizing custom algorithms or software that are central to the research but not yet described in published literature, software must be made available to editors and reviewers. We strongly encourage code deposition in a community repository (e.g. GitHub). See the Nature Portfolio guidelines for submitting code & software for further information.

Data

Policy information about availability of data

All manuscripts must include a data availability statement. This statement should provide the following information, where applicable:

- Accession codes, unique identifiers, or web links for publicly available datasets
- A description of any restrictions on data availability
- For clinical datasets or third party data, please ensure that the statement adheres to our policy

The gene expression data generated during the study are publicly available in the gene expression omnibus (GEO) database under the accession number GSE74391. Survival analyses and immunohistochemistry data are not publicly available to protect patient privacy, but will be made available to authorized researchers who have an approved Institutional Review Board application and have obtained approval from. Please contact the corresponding author with data access requests. All other datasets generated during the study will be made available upon reasonable request to the corresponding author, Dr. Henrik Ditzel, email address: hditzel@health.sdu.dk. Uncropped Western blots are part of the supplementary information.

Field-specific reporting

Please select the one below that is the best fit for your research. If you are not sure, read the appropriate sections before making your selection.

☒ Life sciences ☐ Behavioural & social sciences ☐ Ecological, evolutionary & environmental sciences

For a reference copy of the document with all sections, see nature.com/documents/nr-reporting-summary-flat.pdf

Life sciences study design

All studies must disclose on these points even when the disclosure is negative.

Sample size	*Power calculations were performed to determine that the number of animals and tumor samples should be sufficient to determine significant differences between the different groups.*
Data exclusions	*No data was excluded.*
Replication	*All experiments were performed at least twice, but more often three times. All atempts at replication were successful.*
Randomization	*in vivo: When tumors reached a certain size (indicated in the figure legend), the mice were randomized into treatment groups based on equal tumor sizes.*<br>*in vitro: No randomization was required for in vitro studies.*<br>*clinical samples: Patients treated with AI-monotherapy in the advanced setting (N=109) were selected retrospectively by database extraction from the archives of the Department of Pathology at Odense University Hospital (OUH) from 2014 to 2017, and patients treated with combined CDK4/6i and ET in the advanced setting (N=102) were selected between 2018-2019. The inclusion criteria were ER+ BC patients who had undergone surgery or biopsy for advanced-stage disease treated with either AI-monotherapy or combined CDK4/6i and ET in the advanced setting at OUH, and with available clinical information and pathological verification that the metastatic lesion was of BC origin. Exclusion criteria were insufficient tumor material in the FFPE block or incomplete clinical data.*
Blinding	*Evaluation of the immunohistochemistry staining was performed by a skilled breast pathologist in a blinded setup.*<br>*Evaluation of xenograft tumors volume was not blinded as the same person performed the administration of drugs and evaluated tumor growth.*<br>*No blinding was required for the in vitro studies.*

Behavioural & social sciences study design

All studies must disclose on these points even when the disclosure is negative.

Study description	*Briefly describe the study type including whether data are quantitative, qualitative, or mixed-methods (e.g. qualitative cross-sectional, quantitative experimental, mixed-methods case study).*
Research sample	*State the research sample (e.g. Harvard university undergraduates, villagers in rural India) and provide relevant demographic information (e.g. age, sex) and indicate whether the sample is representative. Provide a rationale for the study sample chosen. For studies involving existing datasets, please describe the dataset and source.*
Sampling strategy	*Describe the sampling procedure (e.g. random, snowball, stratified, convenience). Describe the statistical methods that were used to predetermine sample size OR if no sample-size calculation was performed, describe how sample sizes were chosen and provide a rationale for why these sample sizes are sufficient. For qualitative data, please indicate whether data saturation was considered, and what criteria were used to decide that no further sampling was needed.*
Data collection	*Provide details about the data collection procedure, including the instruments or devices used to record the data (e.g. pen and paper, computer, eye tracker, video or audio equipment) whether anyone was present besides the participant(s) and the researcher, and whether the researcher was blind to experimental condition and/or the study hypothesis during data collection.*
Timing	*Indicate the start and stop dates of data collection. If there is a gap between collection periods, state the dates for each sample cohort.*
Data exclusions	*If no data were excluded from the analyses, state so OR if data were excluded, provide the exact number of exclusions and the rationale behind them, indicating whether exclusion criteria were pre-established.*
Non-participation	*State how many participants dropped out/declined participation and the reason(s) given OR provide response rate OR state that no participants dropped out/declined participation.*
Randomization	*If participants were not allocated into experimental groups, state so OR describe how participants were allocated to groups, and if allocation was not random, describe how covariates were controlled.*

Ecological, evolutionary & environmental sciences study design

All studies must disclose on these points even when the disclosure is negative.

Study description	*Briefly describe the study. For quantitative data include treatment factors and interactions, design structure (e.g. factorial, nested, hierarchical), nature and number of experimental units and replicates.*
Research sample	*Describe the research sample (e.g. a group of tagged Passer domesticus, all Stenocereus thurberi within Organ Pipe Cactus National Monument), and provide a rationale for the sample choice. When relevant, describe the organism taxa, source, sex, age range and any manipulations. State what population the sample is meant to represent when applicable. For studies involving existing datasets, describe the data and its source.*
Sampling strategy	*Note the sampling procedure. Describe the statistical methods that were used to predetermine sample size OR if no sample-size calculation was performed, describe how sample sizes were chosen and provide a rationale for why these sample sizes are sufficient.*
Data collection	*Describe the data collection procedure, including who recorded the data and how.*
Timing and spatial scale	*Indicate the start and stop dates of data collection, noting the frequency and periodicity of sampling and providing a rationale for these choices. If there is a gap between collection periods, state the dates for each sample cohort. Specify the spatial scale from which the data are taken*
Data exclusions	*If no data were excluded from the analyses, state so OR if data were excluded, describe the exclusions and the rationale behind them, indicating whether exclusion criteria were pre-established.*
Reproducibility	*Describe the measures taken to verify the reproducibility of experimental findings. For each experiment, note whether any attempts to repeat the experiment failed OR state that all attempts to repeat the experiment were successful.*
Randomization	*Describe how samples/organisms/participants were allocated into groups. If allocation was not random, describe how covariates were controlled. If this is not relevant to your study, explain why.*
Blinding	*Describe the extent of blinding used during data acquisition and analysis. If blinding was not possible, describe why OR explain why blinding was not relevant to your study.*

Did the study involve field work? ☐ Yes ☐ No

Field work, collection and transport

Field conditions	*Describe the study conditions for field work, providing relevant parameters (e.g. temperature, rainfall).*
Location	*State the location of the sampling or experiment, providing relevant parameters (e.g. latitude and longitude, elevation, water depth).*
Access & import/export	*Describe the efforts you have made to access habitats and to collect and import/export your samples in a responsible manner and in compliance with local, national and international laws, noting any permits that were obtained (give the name of the issuing authority, the date of issue, and any identifying information).*
Disturbance	*Describe any disturbance caused by the study and how it was minimized.*

Reporting for specific materials, systems and methods

We require information from authors about some types of materials, experimental systems and methods used in many studies. Here, indicate whether each material, system or method listed is relevant to your study. If you are not sure if a list item applies to your research, read the appropriate section before selecting a response.

Materials & experimental systems

n/a	Involved in the study	
☐	☒	Antibodies
☐	☒	Eukaryotic cell lines
☒	☐	Palaeontology and archaeology
☐	☒	Animals and other organisms
☐	☒	Human research participants
☒	☐	Clinical data
☒	☐	Dual use research of concern

Methods

n/a	Involved in the study	
☒	☐	ChIP-seq
☐	☒	Flow cytometry
☒	☐	MRI-based neuroimaging

Antibodies

Antibodies used	anti-ER (Thermo Fisher Scientific, RM-9101-S1), phospho-Rb S612 (Thermo Fisher Scientific PA5-12680), anti-CDK6 (abcam, A124821), anti-cyclin A2 (abcam, ab38), anti-p-c-myc S62 (abcam, ab51156), anti-β-actin (abcam, ab6276), anti-cyclin E1 (BD Biosciences, HE12, 551159), anti-P27 (BD Biosciences, 610241), anti-CDK2 (Cell Signaling, 2546), anti-p-CDK2 T160 (Cell Signaling, 2561), anti-cyclin E2 (Cell Signaling, 4132S), anti-cyclin D1 (Cell Signaling, 2978), anti-cyclin D3 (Cell Signaling, 2936), anti-p-Rb S780 (Cell Signaling, 3590), anti-Rb (Cell Signaling, 9309), anti-p-AKT S473 (Cell Signaling, 4060), anti-AKT (pan) (Cell Signaling, 4685), anti-mTOR (Cell Signaling, 2983), anti-p-mTOR S2448 (Cell Signaling, 2971), anti-p-PRAS40 T246 (Cell Signaling, 2997), anti-PRAS40 (Cell Signaling, 2691), anti-p-S6 S235/236 (Cell Signaling, 2211), anti-S6 (Cell Signaling, 2217), anti-cleaved PARP (Cell Signaling, 9541), anti-Bcl-2 (Cell Signaling, 2872), anti-Bax (Cell Signaling, 2772), anti-c-myc (Cell Signaling, D84C12), anti-P21 waf1/cip1 (Santa Cruz, sc-2946), anti-P16 (Santa Cruz, sc-74401), anti-CDK4 (Santa Cruz, sc-23896), anti-GAPDH (Santa Cruz, sc-32233), horseradish peroxidase (HRP)-conjugated goat anti-mouse (Dako, P0447), HRP-conjugated goat anti-rabbit (Dako, P0448).
Validation	All antibodies are commercially available and have been validated by the company for Western blotting and/or immunohistochemistry.

Eukaryotic cell lines

Policy information about cell lines

Cell line source(s)	The original MCF-7and T47D cell lines were obtained from the Breast Cancer Task Force Cell Culture Bank, Mason Research Institute. The original MM-MDA-134 cell line was obtained from the American Type Culture Collection (ATCC).
Authentication	All cell lines were authenticated by Short Tandem Repeat (STR) DNA profiling.
Mycoplasma contamination	All cell lines were tested negative for mycoplasma contamination using Lonza MycoAlert Kit.
Commonly misidentified lines (See ICLAC register)	No commonly misidentified lines were used in this study.

Palaeontology and Archaeology

Specimen provenance	*Provide provenance information for specimens and describe permits that were obtained for the work (including the name of the issuing authority, the date of issue, and any identifying information). Permits should encompass collection and, where applicable, export.*
Specimen deposition	*Indicate where the specimens have been deposited to permit free access by other researchers.*
Dating methods	*If new dates are provided, describe how they were obtained (e.g. collection, storage, sample pretreatment and measurement), where they were obtained (i.e. lab name), the calibration program and the protocol for quality assurance OR state that no new dates are provided.*

☐ Tick this box to confirm that the raw and calibrated dates are available in the paper or in Supplementary Information.

Ethics oversight	*Identify the organization(s) that approved or provided guidance on the study protocol, OR state that no ethical approval or guidance was required and explain why not.*

Note that full information on the approval of the study protocol must also be provided in the manuscript.

Animals and other organisms

Policy information about studies involving animals; ARRIVE guidelines recommended for reporting animal research

Laboratory animals	The recipients were four- to six-week-old female NOG CIEA mice, which were purchased from Taconic. F
Wild animals	No wild animals were used in this study.
Field-collected samples	No field-collected samples were used in this study.
Ethics oversight	For the xenografts, experiments were approved by the Experimental Animal Committee of The Danish Ministry of Justice and were performed at the animal core facility at University of Southern Denmark. For the PDXs, experiments were approved by the Garvan Institute of Medical Research Animal Ethics Committee (protocols 15/25, 18/20 and 18/26).

Note that full information on the approval of the study protocol must also be provided in the manuscript.

Human research participants

Policy information about studies involving human research participants

Population characteristics	Formalin-fixed, paraffin-embedded (FFPE), metastatic lesions of ER+ BC patients treated with AI-monotherapy in the advanced setting (N=109) were selected retrospectively by database extraction from the archives of the Department of Pathology at Odense University Hospital (OUH) from 2014 to 2017. Similarly, patients treated with combined CDK4/6i and ET

	in the advanced setting (N=102) were selected between 2018-2019. Tumors were defined as ER+ if ≥ 1% of the tumor cells were stained positive.
Recruitment	The inclusion criteria were ER+ BC patients who had undergone surgery or biopsy for advanced-stage disease treated with either AI-monotherapy or combined CDK4/6i and ET in the advanced setting at OUH, and with available clinical information and pathological verification that the metastatic lesion was of BC origin. Exclusion criteria were insufficient tumor material in the FFPE block or incomplete clinical data. These criteria yielded 54 metastatic lesions for the AI-monotherapy-treated cohort and 83 metastatic lesions for the combined CDK4/6i and ET cohort. These metastatic samples were taken before commencing treatment with AI-monotherapy or combined CDK4/6i and ET.
Ethics oversight	All selected samples were coded to maintain patient confidentiality. The study was approved by the Ethics committee of the Region of Southern Denmark (approval no. S-2008-0115) and the Danish Data Protection Agency (approval no. 2008-580035(14/10607)).

Note that full information on the approval of the study protocol must also be provided in the manuscript.

Dual use research of concern

Policy information about dual use research of concern

Hazards

Could the accidental, deliberate or reckless misuse of agents or technologies generated in the work, or the application of information presented in the manuscript, pose a threat to:

No	Yes	
☒	☐	Public health
☒	☐	National security
☒	☐	Crops and/or livestock
☒	☐	Ecosystems
☒	☐	Any other significant area

Experiments of concern

Does the work involve any of these experiments of concern:

No	Yes	
☒	☐	Demonstrate how to render a vaccine ineffective
☒	☐	Confer resistance to therapeutically useful antibiotics or antiviral agents
☒	☐	Enhance the virulence of a pathogen or render a nonpathogen virulent
☒	☐	Increase transmissibility of a pathogen
☒	☐	Alter the host range of a pathogen
☒	☐	Enable evasion of diagnostic/detection modalities
☒	☐	Enable the weaponization of a biological agent or toxin
☒	☐	Any other potentially harmful combination of experiments and agents

ChIP-seq

Data deposition

☐ Confirm that both raw and final processed data have been deposited in a public database such as GEO.

☐ Confirm that you have deposited or provided access to graph files (e.g. BED files) for the called peaks.

Data access links<br>*May remain private before publication.*	*For "Initial submission" or "Revised version" documents, provide reviewer access links. For your "Final submission" document, provide a link to the deposited data.*
Files in database submission	*Provide a list of all files available in the database submission.*
Genome browser session<br>(e.g. UCSC)	*Provide a link to an anonymized genome browser session for "Initial submission" and "Revised version" documents only, to enable peer review. Write "no longer applicable" for "Final submission" documents.*

Methodology

Replicates	*Describe the experimental replicates, specifying number, type and replicate agreement.*
Sequencing depth	*Describe the sequencing depth for each experiment, providing the total number of reads, uniquely mapped reads, length of reads and*

Sequencing depth

whether they were paired- or single-end.

Antibodies

Describe the antibodies used for the ChIP-seq experiments; as applicable, provide supplier name, catalog number, clone name, and lot number.

Peak calling parameters

Specify the command line program and parameters used for read mapping and peak calling, including the ChIP, control and index files used.

Data quality

Describe the methods used to ensure data quality in full detail, including how many peaks are at FDR 5% and above 5-fold enrichment.

Software

Describe the software used to collect and analyze the ChIP-seq data. For custom code that has been deposited into a community repository, provide accession details.

Flow Cytometry

Plots

Confirm that:

☒ The axis labels state the marker and fluorochrome used (e.g. CD4-FITC).

☒ The axis scales are clearly visible. Include numbers along axes only for bottom left plot of group (a 'group' is an analysis of identical markers).

☒ All plots are contour plots with outliers or pseudocolor plots.

☒ A numerical value for number of cells or percentage (with statistics) is provided.

Methodology

Sample preparation

BC cells (1 x 106 cells /T75 tissue culture flask) were incubated at 37°C for 24 h and either chemically transfected with specific siRNA at indicated concentrations or treated with the different drugs. Cells were then trypsinized and fixed in ice-cold 70% ethanol. Five hour post fixation, cells were centrifuged, and pellets resuspended in 50µl RNase A (1 mg/ml; Sigma-Aldrich) in PBS. After 30 min incubation at room temperature, cells were stained with propidium iodide (PI, p4170; Sigma-Aldrich) stained and analyzed.

Instrument

Flow cytometer (BD Biosciences)

Software

Flowlogic software (Inivai Technology).

Cell population abundance

For each measurement, 1 x 104 events were collected

Gating strategy

Single cells described on the PI-H to PI-A signal; 2N and 4N populations gated on the basis on propidium iodide signal.

☐ Tick this box to confirm that a figure exemplifying the gating strategy is provided in the Supplementary Information.

Magnetic resonance imaging

Experimental design

Design type

Indicate task or resting state; event-related or block design.

Design specifications

Specify the number of blocks, trials or experimental units per session and/or subject, and specify the length of each trial or block (if trials are blocked) and interval between trials.

Behavioral performance measures

State number and/or type of variables recorded (e.g. correct button press, response time) and what statistics were used to establish that the subjects were performing the task as expected (e.g. mean, range, and/or standard deviation across subjects).

Acquisition

Imaging type(s)

Specify: functional, structural, diffusion, perfusion.

Field strength

Specify in Tesla

Sequence & imaging parameters

Specify the pulse sequence type (gradient echo, spin echo, etc.), imaging type (EPI, spiral, etc.), field of view, matrix size, slice thickness, orientation and TE/TR/flip angle.

Area of acquisition

State whether a whole brain scan was used OR define the area of acquisition, describing how the region was determined.

Diffusion MRI ☐ Used ☐ Not used

Preprocessing

Preprocessing software	*Provide detail on software version and revision number and on specific parameters (model/functions, brain extraction, segmentation, smoothing kernel size, etc.).*
Normalization	*If data were normalized/standardized, describe the approach(es): specify linear or non-linear and define image types used for transformation OR indicate that data were not normalized and explain rationale for lack of normalization.*
Normalization template	*Describe the template used for normalization/transformation, specifying subject space or group standardized space (e.g. original Talairach, MNI305, ICBM152) OR indicate that the data were not normalized.*
Noise and artifact removal	*Describe your procedure(s) for artifact and structured noise removal, specifying motion parameters, tissue signals and physiological signals (heart rate, respiration).*
Volume censoring	*Define your software and/or method and criteria for volume censoring, and state the extent of such censoring.*

Statistical modeling & inference

Model type and settings	*Specify type (mass univariate, multivariate, RSA, predictive, etc.) and describe essential details of the model at the first and second levels (e.g. fixed, random or mixed effects; drift or auto-correlation).*
Effect(s) tested	*Define precise effect in terms of the task or stimulus conditions instead of psychological concepts and indicate whether ANOVA or factorial designs were used.*

Specify type of analysis: ☐ Whole brain ☐ ROI-based ☐ Both

Statistic type for inference<br>(See Eklund et al. 2016)	*Specify voxel-wise or cluster-wise and report all relevant parameters for cluster-wise methods.*
Correction	*Describe the type of correction and how it is obtained for multiple comparisons (e.g. FWE, FDR, permutation or Monte Carlo).*

Models & analysis

n/a	Involved in the study
☐	☐ Functional and/or effective connectivity
☐	☐ Graph analysis
☐	☐ Multivariate modeling or predictive analysis

Functional and/or effective connectivity	*Report the measures of dependence used and the model details (e.g. Pearson correlation, partial correlation, mutual information).*
Graph analysis	*Report the dependent variable and connectivity measure, specifying weighted graph or binarized graph, subject- or group-level, and the global and/or node summaries used (e.g. clustering coefficient, efficiency, etc.).*
Multivariate modeling and predictive analysis	*Specify independent variables, features extraction and dimension reduction, model, training and evaluation metrics.*
